# Elf1 promotes Rad26’s interaction with lesion-arrested Pol II for transcription-coupled repair

**DOI:** 10.1073/pnas.2314245121

**Published:** 2024-01-09

**Authors:** Reta D. Sarsam, Jun Xu, Indrajit Lahiri, Wenzhi Gong, Qingrong Li, Juntaek Oh, Zhen Zhou, Peini Hou, Jenny Chong, Nan Hao, Shisheng Li, Dong Wang, Andres E. Leschziner

**Affiliations:** ^a^Department of Cellular and Molecular Medicine, University of California San Diego, La Jolla, CA 92093; ^b^Division of Pharmaceutical Sciences, Skaggs School of Pharmacy and Pharmaceutical Sciences, University of California San Diego, La Jolla, CA 92093; ^c^Department of Comparative Biomedical Sciences, School of Veterinary Medicine, Louisiana State University, Baton Rouge, LA 70803; ^d^Department of Molecular Biology, School of Biological Sciences, University of California San Diego, La Jolla, CA 92093; ^e^Department of Chemistry and Biochemistry, University of California San Diego, La Jolla, CA 92093

**Keywords:** Cockayne syndrome B, RNA polymerase II, cryo-EM, transcription-coupled repair, Elf1

## Abstract

Transcription-coupled nucleotide excision repair (TC-NER) is a highly conserved DNA repair pathway that removes bulky lesions in the transcribed genome. Cockayne syndrome B protein (CSB), or its yeast ortholog Rad26, has been known for decades to play important roles in the lesion-recognition steps of TC-NER. Another conserved protein ELOF1, or its yeast ortholog Elf1, was recently identified as a core transcription-coupled repair factor. How Rad26 distinguishes between RNA polymerase II (Pol II) stalled at a DNA lesion or other obstacles and what role Elf1 plays in this process remains unknown. Here, we present cryo-EM structures of Pol II-Rad26 complexes stalled at different obstacles that show that Rad26 uses a common mechanism to recognize a stalled Pol II, with additional interactions when Pol II is arrested at a lesion. A cryo-EM structure of lesion-arrested Pol II-Rad26 bound to Elf1 revealed that Elf1 induces further interactions between Rad26 and a lesion-arrested Pol II. Biochemical and genetic data support the importance of the interplay between Elf1 and Rad26 in TC-NER initiation. Together, our results provide important mechanistic insights into how two conserved transcription-coupled repair factors, Rad26/CSB and Elf1/ELOF1, work together at the initial lesion recognition steps of transcription-coupled repair.

## Significance

Transcription-coupled nucleotide excision repair is a conserved repair pathway that deals with bulky lesions that block transcription. It requires coordination of core transcription-coupled repair factors to recognize and distinguish lesion-induced transcription arrest from other non-lesion transcription arrest. This study reports several different states of transcription-coupled repair complexes that are involved in lesion recognition. We also present structure–function studies of lesion-arrested Pol II complexes with two essential transcription-coupled repair factors—CSB/Rad26 and ELOF1/Elf1—and reveal a functional interplay between them during repair that is likely conserved from yeast to human. This study enhances our knowledge of the molecular basis of transcription-coupled repair.

Transcription-coupled DNA nucleotide excision repair (TC-NER), a highly conserved sub-pathway of nucleotide excision repair across all three kingdoms of life, is the first line of defense that detects and removes a broad spectrum of transcription-blocking lesions in the transcribed genome (1–7).

As a master TC-NER factor, Cockayne syndrome group B (CSB) protein, or its ortholog Rad26 in *Saccharomyces cerevisiae*, a member of the Swi2/Snf2 family of nucleosome remodeling helicases/ATPases, plays a crucial early role in eukaryotic TC-NER (1–7). During the lesion recognition steps of TC-NER, CSB/Rad26 distinguishes a lesion-arrested Pol II from other types of arrested Pol II and facilitates subsequent recruitment of downstream repair factors, including CSA, UVSSA, and TFIIH (1, 4–6, 8). In addition to its role in TC-NER, CSB/Rad26 also functions as a processivity factor for Pol II arrested in the absence of DNA damage and regulates a subset of genes crucial for neurological differentiation and development (8–12). Mutations in CSB are linked to Cockayne syndrome, a severe neurodevelopmental disorder characterized by photosensitivity and premature aging (6, 13). Cryo-EM structures have shown that both yeast Rad26 and human CSB bind upstream of a stalled Pol II in an evolutionarily conserved manner (8, 14). While these structures provide important insights into the molecular mechanism of eukaryotic TC-NER, the stalled Pol II complexes were prepared in the absence of DNA lesions. This left an important question unanswered: Does CSB/Rad26 recognize the difference between lesion- and non-lesion-arrested Pol II through different initial interactions?

ELOF1 (human)/Elf1 (yeast *S. cerevisiae* ortholog) was recently identified as another essential transcription-coupled repair factor by several groups using genome-scale CRISPR screens against DNA damaging agents (6, 15, 16). Elf1/ELOF1 is a highly conserved transcription elongation factor that binds to a Pol II elongation complex (15, 17–19). Loss of ELOF1 in humans or deletion of Elf1 in yeast leads to UV sensitivity (6, 15). In human cells, ELOF1 is reported to interact with ubiquitin ligase CRL^CSA^ and promote UVSSA binding to lesion-stalled Pol II. Knocking out ELOF1 leads to a decrease in UV-induced Pol II ubiquitylation and UVSSA monoubiquitylation (6, 16). These findings, however, do not explain the evolutionarily conserved role of Elf1/ELOF1 in TC-NER since yeast lacks counterparts of CRL^CSA^ and UVSSA, and Pol II ubiquitylation is not essential in yeast TC-NER (20).

We set out to establish whether Rad26 uses the same mechanism to recognize all stalled Pol IIs, regardless of the nature of the obstacle, and if and how Rad26 and Elf1 function together, mechanistically, in TC-NER. We report four cryo-EM structures of Pol II stalled at different obstacles, including a UV DNA lesion: a cyclobutene pyrimidine dimer (CPD). These structures reveal that Rad26 uses a common approach to recognize a stalled Pol II but that it interacts with the Rpb4/7 subunits of Pol II in the presence of a lesion. Next, we provide functional evidence supporting a role for Elf1 in promoting the binding of Rad26 to lesion-arrested Pol II. Finally, we present a cryo-EM structure of a lesion-arrested Pol II bound to both Elf and Rad26. Our structure reveals that the presence of Elf1 leads to new interactions between Rad26 and Pol II that are absent from all other Rad26-containing structures. Functional studies highlight the importance of these Elf1-dependent Pol II-Rad26 interfaces in TC-NER. Taken together, these results provide an important mechanistic framework for understanding the functional interplay between two key transcription-coupled repair factors—CSB/Rad26 and ELOF1/Elf1—during TC-NER initiation.

## Results

### Rad26 Has a Common Binding Mode for Different Arrested Pol II Complexes.

**Fig. 1.**
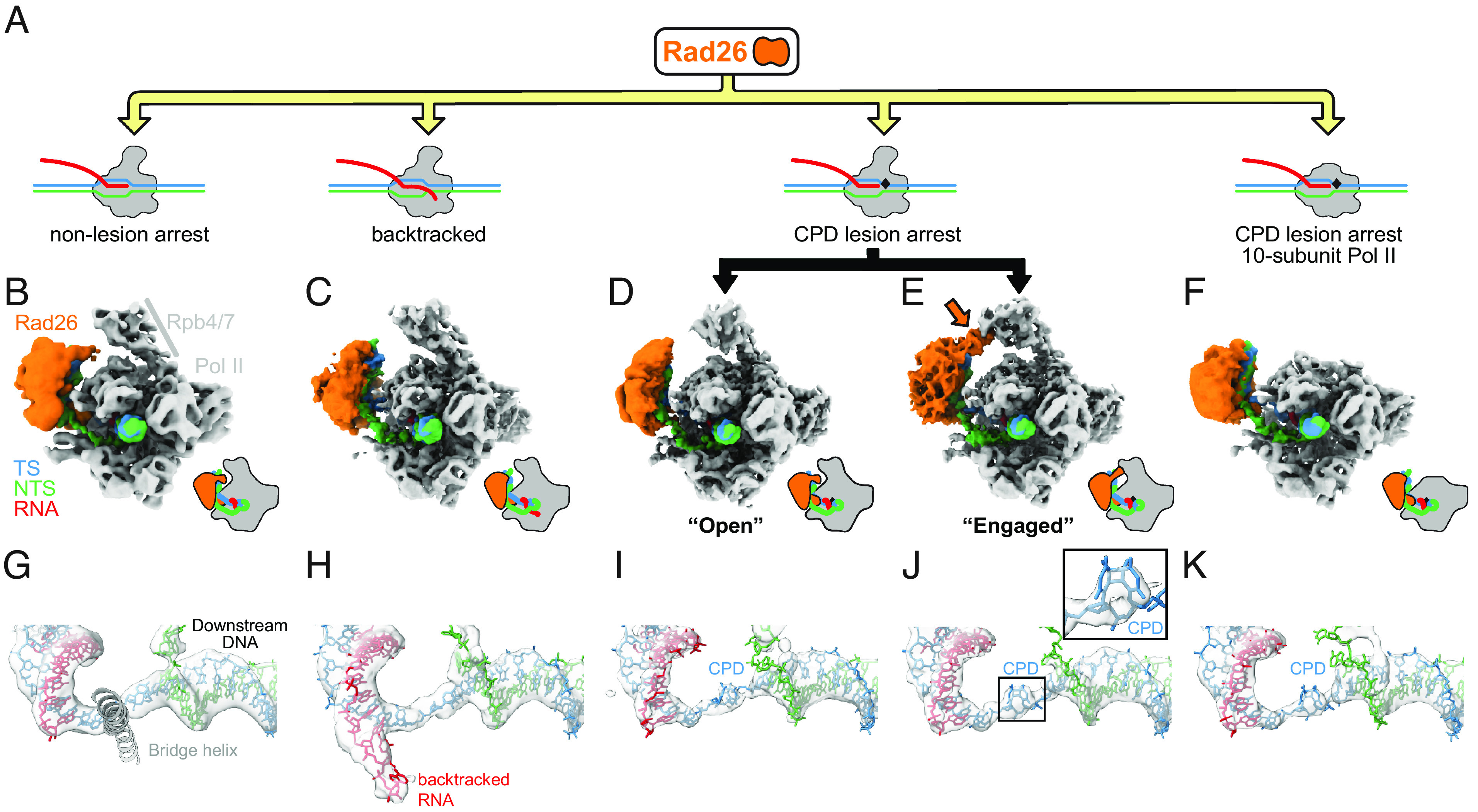
Rad26 interacts in a similar way with paused, CPD-stalled, and Backtracked RNA Pol II. (*A*) Cartoon representation of the different complexes analyzed by cryo-EM. The black rhomboid represents the CPD lesion. (*B*–*F*) Cryo-EM maps of (*B*) Pol II-Rad26 at a non-lesion arrest from our previous work (5.8 Å) (8), (*C*) Backtracked Pol II-Rad26 (4.4Å), (D, E) Pol II(CPD)-Rad26 in states showing either an “Open” (3.7 Å) (*D*), or “Engaged” (3.5 Å) (*E*) interaction between Rad26 and Rpb4/7 (orange arrow), and (*F*) Pol II(CPD)-Rad26 with Pol II lacking Rpb4/7 (4.7 Å). The maps were filtered according to the local resolution and were segmented and colored to highlight the different components, as indicated in (*B*). Cartoon representations of each structure, in the same orientation, are shown next to the maps. (*G*–*K*) Cryo-EM densities corresponding to the DNA/RNA scaffolds in the vicinity of the active site of Pol II segmented from the maps shown in (*B*–*F*). The active site Bridge helix was included as a reference point. A close-up of the cryo-EM density corresponding to the CPD lesion is shown in (*J*). The color scheme used throughout the paper is as follows: Pol II: gray; Rad26: orange; non-template strand: green; template strand: blue; RNA: red.

To investigate the structural basis of Rad26 recognition of lesion-arrested and non-lesion arrested Pol II, we solved cryo-EM structures of Pol II-Rad26 complexes stalled either at a CPD DNA lesion [Pol II(CPD)-Rad26] or containing a transcription scaffold that mimics a backtracked state after arrest at a non-lesion site (Backtracked Pol II-Rad26) (Fig. 1 and *SI Appendix*, Figs. S1–S4). In all structures (Fig. 1 *B–F*), as it was the case in our previous structure of a Pol II-Rad26 complex stalled at a non-lesion site (by nucleotide deprivation, Fig. 1*B*) (8), Rad26 is bound behind the polymerase near the upstream fork of the transcription bubble and interacts with the protrusion and the wall domain of Rpb2, and the clamp coiled-coil of Rpb1. Similarly, the binding of Rad26 bends the upstream DNA by ~80° toward the Pol II stalk (Rpb4/7) in all cases. Thus, Rad26 has a common mode of interacting with Pol II regardless of the type of arrest (Fig. 1).

### An Interaction between Rad26 and Rpb4/7 Is Present in the Lesion-Arrested Pol II-Rad26 Complex.

Although our structures show that Rad26 uses a common binding mode for all arrested Pol II, differences among them point to arrest-specific interactions between Rad26 and Pol II. When we performed three-dimensional classification of the lesion-arrested Pol II-Rad26 complex dataset (Pol II(CPD)-Rad26) (*SI Appendix*, Fig. S1), we found two coexisting conformations. The key difference between them is in the interaction between Rad26 and Rpb4/7 in Pol II: In one state, reminiscent of the structures seen with backtracked and non-lesion-arrested Pol II (Fig. 1 *B* and *C*), and which we termed “Open,” there is no well-defined defined density between Rad26 and Rpb4/7 (Fig. 1*D*); in the second state, Rad26 extends toward and interacts with Rpb4/7 (Fig. 1*E*). We refer to this complex as “Engaged” to reflect this connection between Rad26 and Pol II. This state is specific to the lesion-stalled Pol II(CPD)-Rad26 complex and has three main structural features: Rpb4/7 has shifted toward Rad26 (relative to the core of Pol II); Rad26 has moved toward Rpb4/7, with a concomitant higher bending of the upstream DNA; and, as mentioned above, the density connecting Rpb4/7 and Rad26 is better defined (Fig. 1*E* and *SI Appendix*, Fig. S5 *A–C*). This Rad26-Rpb4/7 interaction and the closer proximity of Rpb4/7 to Rad26 do not appear to be general features of all stalled Pol II complexes as we did not observe them in our previous structures of Pol II-Rad26 stalled at a non-lesion site (8) (*SI Appendix*, Fig. S5*D*) or in our new Backtracked Pol II-Rad26 complex (Fig. 1 *B* and *C* and *SI Appendix*, Fig. S6 *A–C*). In fact, the density between Rad26 and Rpb4/7 is weakest in the Backtracked Pol II-Rad26 state and Rpb4/7 has moved further away from Rad26 (*SI Appendix*, Fig. S6*D*).

The overall architecture of all Rad26-Pol II (arrested) complexes—the binding to and bending of the upstream DNA—is not dependent on the interaction between Rad26 and Rpb4/7: a structure of core Pol II(CPD)-Rad26 with 10-subunit Pol II showed that the DNA was bent to a similar extent in the absence of Rpb4/7 (Fig. 1*F* and *SI Appendix*, Fig. S7). The increased flexibility of Rad26 in this structure (*SI Appendix*, Fig. S7*E*), however, suggests that the interaction of Rad26 with Rpb4/7 stabilizes the former.

### Elf1-Dependent Interactions between Rad26 and a Lesion-Arrested Pol II(CPD).

A previous genome-wide multi-omics analysis of the UV-induced DNA damage response identified human ELOF1 as a top interactor with human CSB (21). Inspired by this, we set out to understand the evolutionarily conserved role of Elf1/ELOF1 in TC-NER at a mechanistic level, focusing on Elf1, the *S. cerevisiae* ortholog of human ELOF1 (*SI Appendix*, Fig. S8 *A* and *B*). We hypothesized that Elf1/ELOF1 could be involved in the initiation of TC-NER by modulating the interaction between Pol II and Rad26/CSB.

**Fig. 2.**
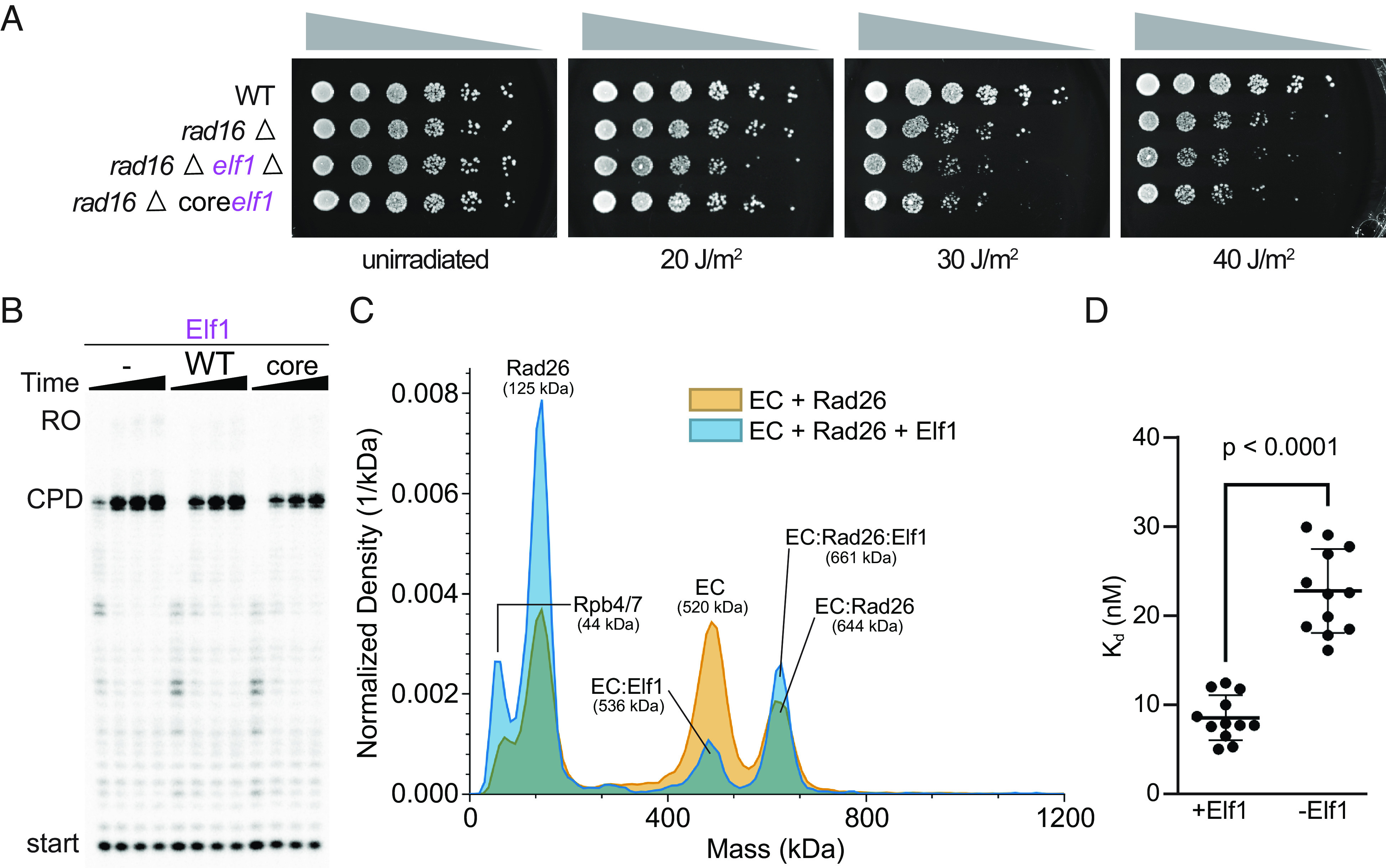
Elf1 enhances interactions between Pol II and Rad26. (*A*) Deletion of Elf1 leads to UV sensitivity and can be rescued by Elf1core. (*B*) Elf1, or Elf1core have no effect on the stalling of Pol II at a CPD lesion. Time points were 0.3, 1, 3, and 10 min. (*C*) Mass photometry of EC + Rad26 in the absence or presence of Elf1. Peak identities and predicted molecular weights are indicated. (*D*) K_d_s for the interaction between Pol II (EC) stalled at a CPD lesion and Rad26 in the absence or presence of Elf1 were determined from multiple repeats of the experiment in (*C*). *P* < 0.0001, two-tailed Student’s *t* test. Data are mean and SD (n = 12). The final Elf1 concentrations were 1 μM (*B*) and 0.5 μM (*C*). All assays were performed at least three times independently.

Yeast Elf1 and human ELOF1 share a highly conserved core domain (*SI Appendix*, Fig. S8*A*). In addition, Elf1 contains an intrinsically disordered, yeast-specific C-terminus (*SI Appendix*, Fig. S8 *A* and *B*), which was not observed in a published structure of Pol II-Spt4/5-Elf1 even though full-length protein was used (19). A yeast strain containing a “core” Elf1 was created by introducing an early stop codon at amino 86 (creating a C-terminal truncation) to mimic human ELOF1 (*SI Appendix*, Fig. S8 *A* and *B*). This strain with core Elf1 protein (rad16ΔElf1core) behaved similarly in its response to UV damage compared with the yeast strain (rad16Δ) with full-length Elf1 protein (Fig. 2*A*).

Given what we observed in vivo, we tested whether Elf1 (WT) or Elf1core proteins behaved in similar ways in vitro. We first measured their effect on the stalling of Pol II at a CPD lesion in a transcription assay. The stalling pattern of Pol II was the same with either construct (Fig. 2*B*). Next, we used mass photometry to measure the affinity between a lesion-arrested Pol II and Rad26 in the absence or presence of Elf1. Intriguingly, we found that Elf1 promoted the formation of a lesion-arrested Pol II(CPD)-Rad26 complex, with the K_d_ for the Pol II(CPD)-Rad26 interaction shifting from 23 ± 5 nM to 9 ± 3 nM in the presence of Elf1 (Fig. 2 *C* and *D* and *SI Appendix*, Fig. S9).

**Fig. 3.**
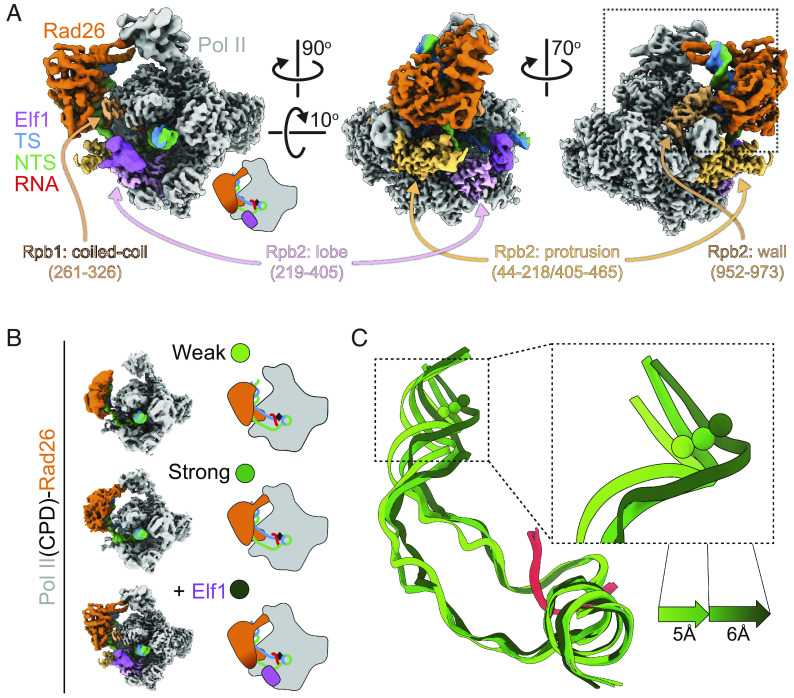
CryoEM structure of Pol II(CPD)-Rad26-Elf1 complex. (*A*) 3.1Å cryo-EM map of the Pol II(CPD)-Rad26-Elf1 complex, with Elf1 colored in light purple. The regions of Pol II that interact with Elf1 or Rad26 are colored in shades of purple or orange, respectively. (*B, C*) The increase in the bend angle of the upstream DNA mirrors the improvement of the Rad26 density in the cryo-EM maps. (*B*) Cartoon representations of the three structures being compared. (*C*) The DNA/RNA scaffolds were superimposed using the downstream DNA and color-coded as shown on the left. The shifts in the upstream DNA between these states are shown.

**Fig. 4.**
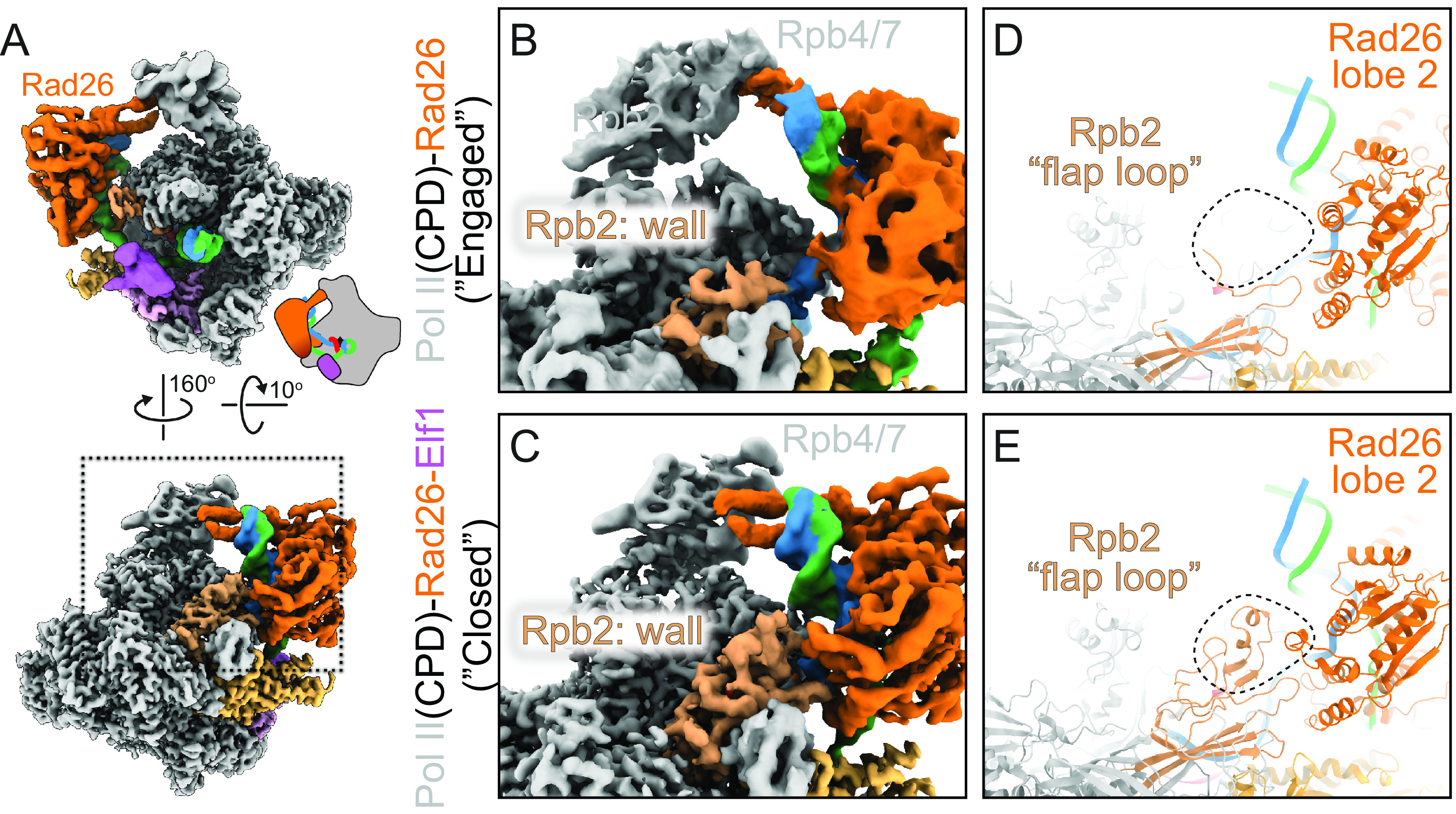
New interaction interfaces between Pol II and Rad26 observed in the Pol II(CPD)-Rad26-Elf1 complex. (*A*) Overview of Pol II(CPD)-Rad26-Elf1 complex. (*B*–*E*) Zoomed-in views of the area highlighted by the square in (*A*) for Pol II(CPD)-Rad26 without (*B* and *D*) or with (*C* and *E*) Elf1 bound. (*B* and *C*): Cryo-EM densities. (*D* and *E*): Corresponding models. The dashed square highlights the region where new density is present in the Pol II(CPD)-Rad26-Elf1 structure (*E*). The structural elements that become ordered in both Pol II and Rad26 are shown in (*E*).

To understand the structural basis of Elf1’s role in promoting the interaction between Rad26 and Pol II stalled at a CPD lesion, we solved a cryo-EM structure of a Pol II(CPD)-Rad26-Elf1 complex (Fig. 3*A* and *SI Appendix*, Figs. S10 and S11 *A–G*). Elf1 is bound in the downstream channel, next to the lobe domain of Rpb2 and bridging the cleft, as previously reported (*SI Appendix*, Fig. S8 *C–E*) (18, 19). The presence of Elf1 resulted in a significant improvement in the local resolution of Rad26 to 4Å, from 8Å in the “Engaged” state, our second-best map (*SI Appendix*, Fig. S11*A*). This stabilization effect is likely through a long-range allosteric path (Elf1 ↔ Pol II protrusion domain ↔ Pol II wall domain ↔ Rad26), as there are no direct interactions between Rad26 and Elf1 (Fig. 3*A*). Most strikingly, the Pol II(CPD)-Rad26-Elf1 complex, which we refer to here as the “Closed” state, showed new density at the interface between lobe 2 of Rad26 and the wall domain of Rpb2, corresponding to interfaces absent from any of the other 5 Rad26-Pol II complex structures we have solved to date (Fig. 4 and *SI Appendix*, Fig. S11 *H* and *I*). The flap-loop of Rpb2, which was disordered in the other structures, is folded, and interacts directly with a short loop–helix–loop region in the Rad26 lobe 2 (631 to 644aa, which was also disordered in previous structure 5VVR) (Fig. 4). This newly folded short loop–helix–loop region is next to the conserved HD-2-1 motif of Rad26, which inserts in the upstream fork of the DNA transcription bubble. The interaction between Rad26 and Rpb4/7 seen in the “Engaged” state is preserved in this structure but, as noted above, is better defined due to the higher resolution of the cryo-EM map (Figs. 3 and 4). Comparing the bending of the upstream DNA among the “Open”, “Engaged”, and “Closed” states highlights how Rad26 (and the DNA to which it is bound) shifts toward Pol II as more and/or better-defined interactions between them are seen in the maps (Fig. 3 *B* and *C*). This is consistent with the idea that these structures represent steps toward the assembly of a lesion-stalled Pol II-Rad26-Elf1 complex for TC-NER.

### Pol II-Rad26 Interactions Stabilized by Elf1 Are Required for Their Functional Coupling.

The resolution of the Pol II(CPD)-Rad26-Elf1 complex allowed us to identify key residues involved in the Elf1-induced Pol II-Rad26 protein interfaces revealed by this structure. One interface (Interface A) is located between a short loop–helix–loop motif of Rad26 lobe 2 (631 to 644aa, next to HD-2-1 motif) and Pol II Rpb2 wall/flap loop domain. The other interface (Interface B) is between Rad26 [475 to 490aa, connecting conserved motifs IIa (switch) and III in lobe 1] and Rpb1 Clamp coiled-coil domain. To further understand the functional significance of these interfaces, we mutated several conserved residues expected to disrupt them: Rad26(R635D/K638D/R639D) (“RKR/DDD”) and Rad26(L483A/K486A/K487A) (“LKK/AAA”). Rad26-RKR/DDD should disrupt the interface between Rad26 and Pol II Rpb2 wall/flap loop domain (Interface A) (Figs. 3*A* and 4), while Rad26-LKK/AAA should disrupt the interface between Rad26 and Pol II Rpb1 Clamp coiled-coil domain (Interface B) (Fig. 3*A*).

**Fig. 5.**
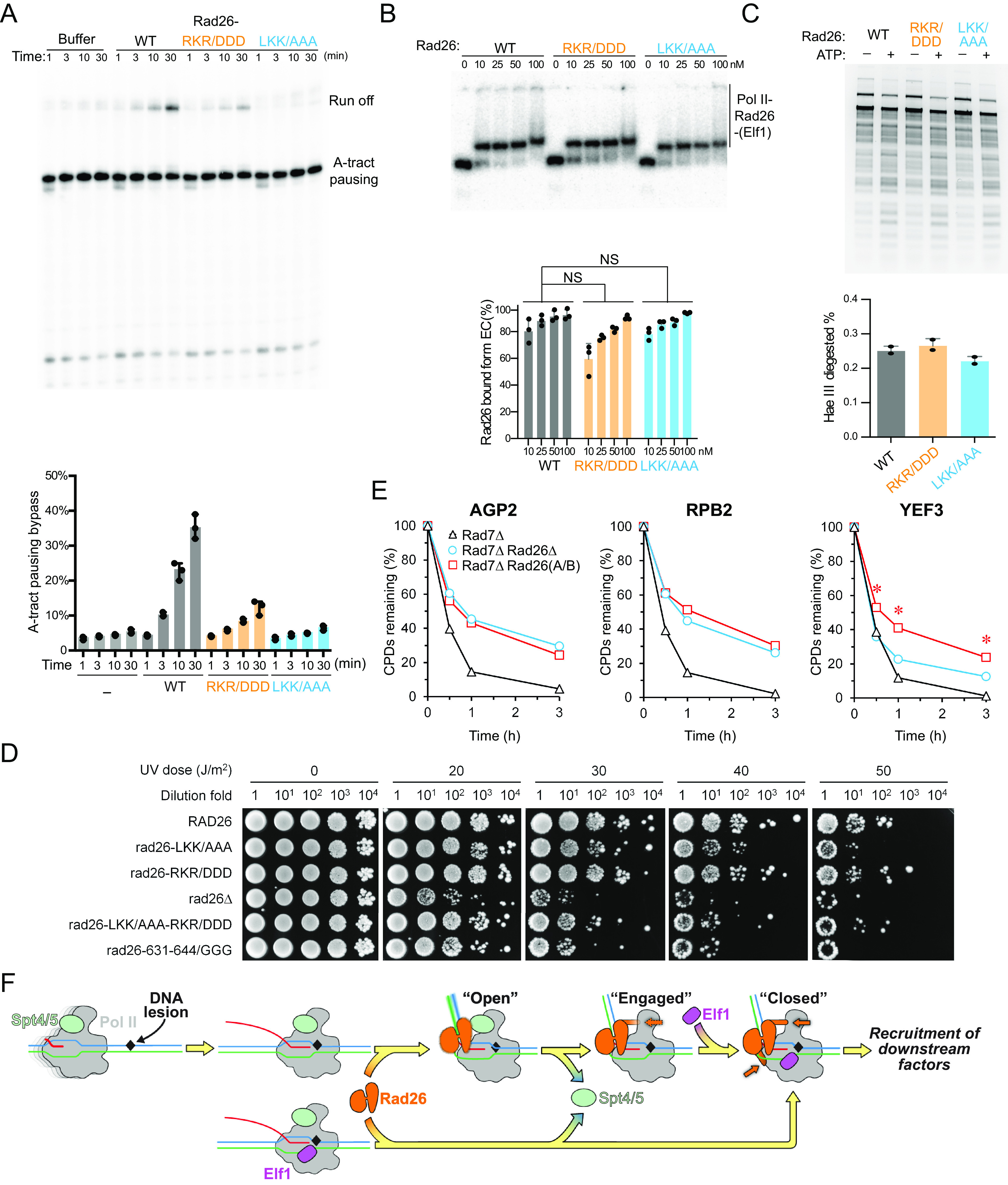
Functional significance of Pol II-Rad26 interface in TC-NER. (*A*) Rad26 mutants (Rad26-LKK/AAA and Rad26-RKR/DDD) are impaired in promoting Pol II transcription bypass of a pausing sequence. (*B*) Rad26-LKK/AAA and Rad26-RKR/DDD do not have impaired binding to Pol II. (*C*) Rad26-LKK/AAA and Rad26-RKR/DDD do not have impaired ATP-dependent DNA translocase activity. All assays in (*A*–*C*) were performed at least three times independently. (*D*) Effects of Rad26 mutations on UV sensitivity. Images are from plates spotted with yeast cells of indicated Rad26 mutations following irradiation with the indicated UV doses. (*E*) Plots showing the effect of *rad26-LKK/AAA-RKR/DDD* on TC-NER. The means of percent CPDs remaining at all CPD sites 50 nucleotides downstream of the major transcription start site (TSS) in the template strand of *AGP2*, *RPB2,* and *YEF3* gens in the indicated cells are plotted. Asterisks (*) indicates that the percent CPDs remaining in the *rad7Δ rad26-LKK/AAA-RKR/DDD* cells is significantly different from that in the *rad7Δ rad26Δ* cells at the corresponding repair time points (*P* values < 10^−10^, Student’s *t* test). (*F*) Stepwise model for lesion recognition and reconfiguration during TC-NER initiation in yeast. The color scheme used in Fig. 5*F* is as follows: Elf1: magenta, Spt4/5: lime. Other colors are the same as in Fig. 1.

We previously showed that Rad26 improves transcription-coupled lesion recognition fidelity and rescues Pol II from non-lesion arrests (8, 11). This relies on coupling Rad26’s ATP-dependent DNA translocase activity with Pol II’s forward translocation to promote Pol II bypass of non-lesion-induced arrests. To test whether the Rad26-Pol II interfaces we identified in the Pol II(CPD)-Rad26-Elf1 structure are necessary for this function, we purified Rad26-LKK/AAA (Interface B mutant) and Rad26-RKR/DDD (Interface A mutant) and tested their ability to promote Pol II bypass of a pausing sequence. As shown in Fig. 5*A*, both Rad26 mutants were significantly impaired in this assay. Importantly, this effect is not due to a reduction in Rad26’s binding to Pol II: Both Rad26-RKR/DDD and Rad26-LKK/AAA bind to Pol II with affinities equivalent to that of Rad26(WT) (Fig. 5*B*). Fig. 5*B* also shows that the concentrations of Rad26 proteins used in the transcription bypass assay in Fig. 5*A* (200 nM) were saturating, ruling out the possibility that the effects seen were due to reduced binding of certain Rad26 mutants to Pol II. Another possible explanation for the differences observed in Fig. 5*A* was an effect of the mutants on the ATP-dependent DNA translocase activity of Rad26. To test this, we generated and purified the RKR/DDD and LKK/AAA mutants in the context of a constitute-active form of Rad26 (a deletion of the N-terminal auto-inhibitory motif) and characterized their translocase activities using a restriction enzyme accessibility assay with nucleosomes as substrates. All three constructs behaved similarly in this assay (Fig. 5*C*), showing that the effects of the mutations on Rad26’s ability to help Pol II bypass a pausing sequence were not due to different translocation activities. Taken together, these results support a functional role for the Rad26-Pol II interfaces (Interfaces A and B) identified here in coupling Rad26 and Pol II activities for discriminating between lesions and non-lesions.

### Mutations of Pol II-Rad26 Interfaces Abolish TC-NER Activity In Vivo.

To test the in vivo role of these interfaces in TC-NER, we first measured the UV sensitivity of the mutants we had designed to disrupt them. The highly efficient Global Genome (GG)-NER in yeast cells would mask the effects of Rad26 on UV sensitivity and TC-NER in cells (22). We therefore used GG-NER-defective *rad7Δ* cells for our assays. As shown in Fig. 5*D*, all strains with mutations in Interfaces A and/or B had increased UV sensitivity compared with Rad26(WT), indicating that these mutations render Rad26 TC-NER defective. Strikingly, we found that three mutations (among all the strains we tested) are most deleterious, increasing UV sensitivity to a level approaching that seen in a *rad26* deletion strain: rad26-LKK/AAA; rad26-LKK/AAA-RKR/DDD; or the strain replacing the loop–helix–loop of Rad26 lobe 2 at Interface A with GGG (rad26-631-644/GGG) (Fig. 5*D*).

To further characterize the role of the Rad26-Pol II interactions identified here in TC-NER, we used a well-established TC-NER assay (23) that can quantitatively measure the kinetics of TC-NER at different genomic loci at base resolution. We analyzed the effects of Rad26 interface mutations on TCR in three representative genomic loci (*AGP2*, *RPB2,* and *YEF3*), which are transcribed at low, moderate, and high rates, respectively (22, 24). In *rad7Δ* cells expressing wild-type Rad26, rapid repair of CPDs can be seen immediately downstream of the major transcription start site (TSS) in the template strand (TS) of these genes (Fig. 5*E*, black curve, and *SI Appendix*, Fig. S12), indicating rapid TC-NER. TC-NER was slow in *rad7Δ rad26Δ* cells (Fig. 5*E* and *SI Appendix*, Fig. S12), especially in the region over 50 nucleotides downstream of the transcription start sites (TSS) (*SI Appendix*, Fig. S12), where the RNA Pol II switches to transcription elongation mode and TC-NER is repressed by Spt4/5 in the absence of Rad26 (22). *Rad7Δ* cells expressing a Rad26 mutated on both interfaces (*rad7Δ* rad26-LKK/AAA-RKR/DDD) and *rad7Δ* cells lacking Rad26 (*rad7Δ rad26Δ*) showed similar rate of TC-NER (slow) in the AGP2 and RPB2 genes (Fig. 5*E*, compare cyan and red curves, and *SI Appendix*, Fig. S12), indicating that the Rad26 double-interface mutant (rad26-LKK/AAA-RKR/DDD) has no Rad26-dependent TC-NER activity in these genes. Interestingly, the TC-NER rate in the *YEF3* gene was even slower, in a significant way, in *rad7Δ* rad26-LKK/AAA-RKR/DDD cells than in *rad7Δ rad26Δ* cells (Fig. 5*E*, compare red and cyan curves, and *SI Appendix*, Fig. S12). This suggests that the presence of the Rad26 double-interface mutant (rad26-LKK/AAA-RKR/DDD) has a dominant negative effect on TC-NER in the rapidly transcribed gene. Taken together, our in vitro and in vivo functional data support an important role for the Rad26-Pol II interactions we identified in the Pol II(CPD)-Rad26-Elf1 complexes in TC-NER as well as in coupling the activities of Rad26 and Pol II to allow for the control of lesion recognition/discrimination and the transcriptional bypass of non-lesion arrests.

## Discussion

Here, we report several cryo-EM structures of Pol II-Rad26 complexes arrested at lesion and non-lesion obstacles, revealing three different states: 1) an initial, common binding mode between Rad26 and any arrested Pol II, the “Open” state, characterized by binding to and bending of the upstream DNA, and the absence of a well-defined interaction between Rad26 and Rpb4/7; 2) a lesion-specific “Engaged” state with a well-defined interaction between Rad26 and Rpb4/7 in Pol II; and 3) a “Closed” state, promoted by Elf1, where new interfaces form between Rad26 and Pol II.

### The “Open” State Represents the Initial Recruitment of Rad26 to Arrested Pol II.

A common binding mode of Rad26 to different forms of arrested Pol II is consistent with the dual roles of Rad26 in repair (improving lesion recognition fidelity) and transcription elongation. The “Open” state represents the initial recruitment of Rad26 to an arrested Pol II, regardless of the nature of that arrest. At this step, Rad26 is recruited to the upstream DNA fork of arrested Pol II, where it inserts its conserved W752 at the upstream edge of the transcription bubble. Rad26 then uses its ATP-dependent translocase activity to track along the template strand in a 3′-5′ direction, moving toward Pol II. If Pol II is arrested at a non-lesion or small lesion transcription barrier, Rad26’s translocation can promote Pol II forward translocation and eventually obstacle bypass. If, instead, Pol II is arrested at a bulky lesion, bypass cannot occur and the interaction between Rad26 and Pol II’s Rpb4/7 becomes stabilized, leading to the “Engaged” state. Recruitment of Elf1 further stabilizes the Rad26-Pol II interactions, including the development of interfaces not seen in its absence, to form the “Closed” state. Note that the Rbp4/7 region is also a major docking site for the elongation factor Spt4/5 complex; it therefore seems reasonable to speculate that the conformational changes involved in the transition of a CPD-lesion stalled Pol II-Rad26 from “Open” to “Engaged” and “Closed” may lead to the complete displacement of Spt4/5. The ultimate consequence of these structural changes is the recruitment of downstream repair factors, such as TFIIH.

Recently, a lower resolution cryo-EM structure of *Komagataella pastoris* Pol II-Rad26-Elf1 elongation complex on a nucleosome template was published (PDB 8HE5) (25). Although the authors found that Rad26 binds upstream of Pol II, the conformation of *K. pastoris* Pol Rad26 is different from that in the previously reported yeast Pol II-Rad26 and human Pol II-CSB complexes, as well as in the structures presented in this manuscript. The interactions between Pol II and Rad26 are less extensive and may therefore represent an early intermediate preceding the “Open” state described here.

### A Lesion Arrest Leads to a Defined Interaction between Rad26 and Pol II’s Rpb4/7 “stalk”.

Our structural analysis revealed that a well-defined interaction is formed between Rad26 and the Pol II stalk (Rpb4/7) when Pol II is arrested at a CPD lesion (the “Engaged” state) (Fig. 1*E*). Rpb4/7 plays an important role in controlling the conformational dynamics of the Pol II clamp and is a hub for interactions with several transcription factors, including Spt4/5 and Spt6. As a result, Rpb4/7 plays important roles in several molecular and cellular processes, including transcription and DNA repair (18, 26). A previous genetic study in *S. cerevisiae* showed that Rpb4/7 promotes Rad26-dependent TC-NER while suppressing Rad26-independent TC-NER (27). On the other hand, Spt4/5, an elongation factor that binds both Rpb4/7 and the protrusion domain of Pol II (18, 19, 28), functions as an inhibitor of TC-NER (29). We propose that the steric exclusion of Spt4/5 by the lesion-induced stabilization of the interaction between Rad26 and Rpb4/7 is a major step in committing a lesion-stalled Pol II to TC-NER. Furthermore, a previous computational study suggested that tethering Rad26-NTD with Pol II Rpb4/7 is important for Pol II-Rad26 complex assembly and plays a key role in anchoring Rad26 to Pol II and establishing a productive orientation with respect to the transcription bubble (30).

### Binding of Elf1 Promotes the Formation of Rad26-Pol II Interactions Critical for TC-NER.

Addition of Elf1 increased the affinity of Rad26 for Pol II in vitro and resulted in new interactions between Rad26 and Pol II seen in the cryo-EM structure of the Rad26-Pol II(CPD)-Elf1 complex that were absent from all other CPD-stalled structures we have solved to date. Increased affinity is also consistent with the significant improvement in the density for Rad26 in the structure. Given the absence of direct interactions between Elf1 and Rad26, we propose that Elf1 exerts its effect on the Rad26-Pol II interaction through long-range allostery: binding of Elf1 to Pol II encircles the downstream DNA within the cleft and reduces the conformational dynamics of the Pol II clamp, in turn stabilizing the interfaces between Rad26 and Pol II. This idea is supported by predictions from a dynamic network analysis (30).

The Pol II-Rad26 interfaces induced by Elf1 are critical for TC-NER; disrupting these interfaces completely abolished TC-NER in vivo (Fig. 5*E*). Interestingly, at the highly transcribed *YEF3* gene, we found that a Rad26-Pol II interface mutant led to even slower TC-NER than the cells with deletion Rad26 (Fig. 5*E*), suggesting that the Rad26-Pol II interface mutant protein not only affects Rad26-dependent TC-NER, but also sterically blocks Rad26-independent TC-NER at certain gene loci. Taken together, our data suggest that the Pol II(CPD)-Rad26-Elf1 complex represents the fully assembled complex in TC-NER that is committed for recruitment of downstream repair factors, such as TFIIH.

### Conservation and Differences between Yeast and Human TC-NER Initiation.

Core TC-NER factors are highly functionally and structurally conserved between yeast and humans. Indeed, previous studies revealed striking structural similarity between yeast Pol II-Rad26 and human Pol II-CSB complexes as well as their mechanisms (8, 14). Our study sheds important mechanistic insights into the conserved roles of TC-NER factors Rad26/CSB and Elf1/ELOF1 for TC-NER initiation. Considering the data presented here, along with previous work, we propose a stepwise model for the initiation of TC-NER (Fig. 5*F*). Rad26 first binds to the upstream of a stalled Pol II in an “Open” state, regardless the nature of the obstacle. In this state, Rad26 binds at the upstream end of the transcription bubble and the initial interaction of Rad26 with an arrested Pol II results in bending of the upstream DNA. Rad26’s remodeler-like DNA translocation biases Pol II forward, promoting the bypass of non-lesion obstacles or small lesions (8, 11). In this scenario, Pol II may resume productive transcription elongation after obstacle bypass and Spt4/5 may remain partially associated with Pol II (presumably via its interaction with Rpb4/7). In the case of a Pol II arrested at a bulky lesion, the interaction between Rad26 and Rpb4/7 becomes more defined (“Engaged” state). The presence of Elf1 induces additional interactions between Rad26 and Pol II (“Closed” state). In the “Engaged” and “Closed” states, the interactions between Rad26 and Rpb4/7 lead to the displacement of Spt4/5. The conformational changes from “Open” to “Engaged” to “Closed” set the stage for the recruitment of downstream factors, such as TFIIH and XPA, for TC-NER (14, 25, 31, 32). These initial steps are likely conserved between yeast and humans, though additional factors and layers in human cells are involved in regulating TC-NER. For example, CSA and UVSSA, for which there are no yeast counterparts, are important for regulating Pol II ubiquitylation and in turn recruitment of TFIIH (33). During TC-NER, Pol II needs to be displaced from the lesion site to allow lesion exposure to downstream repair factors. A remaining question is when and how Pol II is displaced from a lesion site during TC-NER. Future studies will focus on elucidating the molecular mechanism of Pol II displacement during TC-NER and dissect the potential roles of individual repair factor, such as Rad26, Elf1, TFIIH, and XPA, in this critical process.

## Materials and Methods

### Protein Expression and Purification.

Expression and purification of Rad26 were performed essentially as previously described (8). Briefly, recombinant Rad26 protein was expressed in *Escherichia coli* strain Rosetta 2(DE3) (Novagen) and purified by Ni-NTA agarose (Qiagen), Hi-Trap Heparin HP (GE Healthcare), and Superdex 200 10/300 GL columns (GE Healthcare). Rad26 mutants were purified in the same manner as wild-type proteins. Expression and purification of yeast TFIIS were performed as described (34). Expression and purification of yeast Elf1 and yeast Elf1core were performed essentially as previously described (18, 35). Briefly, GST-tagged Elf1 protein was expressed in *E. coli* strain Rosetta 2(DE3) (Novagen) and purified by Glutathione Sepharose 4 Fast Flow resin (GE Healthcare), and Superdex 200 10/300 GL column (GE Healthcare). Elf1core was purified in the same manner as wild-type protein. Recombinant Spt4/5 was expressed and purified as described (36).

*S. cerevisiae* 10-subunit Pol II was purified essentially as previously described (37). Briefly, Pol II (with a protein A tag in the Rpb3 subunit) was purified by an IgG affinity column (GE Healthcare), followed by Hi-Trap Heparin (GE Healthcare) and Mono Q anion exchange chromatography columns (GE Healthcare). Pol II was purified by incubating 10-subunit Pol II with threefold of Rpb4/7 followed by gel filtration. His6-tagged Rpb4/7 heterodimer was purified from *E. coli* by Ni-affinity chromatography followed by gel filtration as previously described (38).

### In Vitro Transcription Assay.

Pol II elongation complexes were assembled essentially as previously described with a labeled RNA primer (8). For transcription assay to test the effect of Elf1 or Rad26, purified Elf1 (300 nM or 1 µM) or Rad26 proteins (100 or 200 nM) were also included in transcription assays. In vitro transcription was started by adding rNTPs mixture to a final concentration of 1 mM each and quenched at different time points. The reacted samples were boiled for 10 min at 95 °C in formamide loading buffer, and the RNA transcripts were separated by denaturing PAGE (6M urea). The gel was visualized by phosphorimaging and quantified using Image Lab software (Bio-Rad).

### Preparation of Pol II-Rad26 and Pol II(CPD)-Rad26-Elf1 Complexes for Electron Microscopy.

Template and non-template DNA oligonucleotides were obtained from IDT and further purified by PAGE. PAGE-purified RNA oligonucleotides were purchased from Dharmacon. HPLC-purified CPD lesion-containing template was purchased from TriLink. The RNA, template DNA (non-damaged or CPD lesion containing) and non-template DNA were annealed to form the scaffold as previously described (8).

To form the CPD-arrested Pol II complex, Pol II and threefold excess of scaffold were mixed and further purified by gel filtration in 50 mM HEPES, pH 7.4, 5 mM DTT, 5 mM MgCl_2_, and 40 mM KCl. To form the backtracked Pol II complex, Pol II and the scaffold were incubated in 50 mM HEPES, pH 7.4, 5 mM DTT, 5 mM MgCl_2_, and 40 mM KCl. To form the backtracked Pol II-Rad26 complex, Rad26 was added to backtracked Pol II complex and incubated for 30 min. The final buffer was composed of 50 mM HEPES, pH 7.4, 5 mM DTT, 5 mM MgCl_2_, 40 mM KCl, and 200 mM NaCl. For the Pol II(CPD)-Rad26 complex, a final concentration of 0.02% glutaraldehyde was added after adding Rad26 and incubated for another 30 min. The crosslink reaction was terminated by adding 1M Tris-HCl, pH 8.0 to a final concentration of 100mM. The final concentrations of the different components were 1 μM Pol II, 2 μM Rad26, and 1.1 μM scaffold. To form the Pol II(CPD)-Rad26-Elf1 complexes, fourfold excess of Elf1 was incubated with Pol II(CPD)-Rad26 complex. The final concentrations of the different components were 1 μM Pol II, 2 μM Rad26, 1.1 μM scaffold, and 5 μM Elf1.

The sequences used for elongation complex preparation are as follows: non-template DNA, 5′-CTAGTTGATCTCATATTTCATTCCTACTCAGGAGAAGGAGCAGAGCG-3′; template DNA, 5′-CGCTCTGCTCCTTCTCCCATCCTCTCGATGGCTATGAGATCAACTAG-3′; CPD lesion-containing template DNA, 5′-CGCTCTGCTC CTTCTCCXXTCCTCTCGATGGCTATGAGATCAACTAG-3′ (XX = CPD lesion); RNA [for Pol II(CPD)], 5′-AUCGAGAGGA-3′; RNA (for Backtracked Pol II), 5′-AUCGAGAGGAUGCAGAC-3′.

### Measure Kd Values of Rad26 and CPD-Stalled Pol II Complex in the Presence or Absence of Elf1.

The 12-subunit CPD-arrested Pol II complex (EC) was prepared in a same manner as described in the above section. The 12-subunit CPD-arrested Pol II complex was then mixed with an equimolar amount of Rad26. The mixture was then diluted to different final concentrations. For the Kd measurement in the presence of Elf1, additional Elf1 (final 500 nM) was included in the system. The mixture was incubated for 20 min to reach equilibrium before being loaded to the Mass Photometer (Refeyn Two^MP^) to measure Kd values. The Kd measurement was performed on Mass Photometry referring to the manufacturer’s protocol (Refeyn) and previous publication (39). For each case, we measure Kd values at four different concentrations [for Kd measurement without Elf1, the Kd values were measured at four different final concentrations of 12-subunit CPD-arrested Pol II complex (5, 10, 15, and 25 nM, respectively); for Kd measurement with Elf1, the Kd values were measured at four different final concentrations (2, 5, 15, and 20 nM, respectively)] and each concentration are measured in three independently (total 12 measures). For each test, software of AcquireMP 2.3.0 was used for data collection. First, 20 μL sample was loaded to the microscope. Then, after focus finding by buffer-free mode, a 1-min video was recorded. For data analysis, the movies were loaded to the software Refeyn DiscoverMP 2.3.0 for mass identification and particle counting. In the resulting plot, the Rad26, EC, and complex EC•Rad26 were isolated to three peaks with three different molecular weight. The counts of each peak on a range of 100 kDa were extracted for further analysis. For the Kd calculation, a conversion factor (f_conversion_) was introduced to transform the particle counts to molar concentration of each protein. The Kd values were calculated using the following formula. Kd values and their SDs were calculated from values from 12 reads.

$$f_{\text{conversion}}=\frac{\left[Rad26\right]\text{initial}\left[EC\right]\text{initial}}{\left(\text{CountsRad}26+\text{CountsEC}+2\cdot \text{Counts}\left(\text{EC}•\text{Rad}26\right)\right)},$$

$$\left[\text{EC}•\text{Rad}26\right]=f{}_{\text{conversion}}\;\cdot \text{Counts}\left(\text{EC}•\text{Rad}26\right),$$

$$\left[EC\right]=f_{\text{conversion}}\;\cdot \text{Counts}\text{EC},$$

$$\left[\text{Rad}26\right]=f_{\text{conversion}}\cdot \text{CountsRad}26,$$

$$\text{Kd}=\frac{\left[\text{Rad}26\right]\;\left[\text{EC}\right]\;}{\left[\text{EC}•\text{Rad}26\right]}.$$

### Electron Microscopy.

An aliquot of 4 µL of each sample was applied to glow-discharged Quantifoil holey carbon films R1.2/1.3 Cu grids. The grids were blotted and plunge-frozen in liquid ethane using a Vitrobot Mark IV (FEI). Data collection was performed using Leginon (40) on an FEI Talos Arctica operated at 200 kV, equipped with a Gatan K2 summit direct detector. For the Pol II(CPD)-Rad26 sample, 3,358 movies were recorded in counting mode at a dose rate of 11.3 electrons pixel^−1^s^−1^ with a total exposure time of 7.05 s sub-divided into 150 ms frames, for a total of 47 frames. The images were recorded at a nominal magnification of 36,000× resulting in an object-level pixel size of 1.16 Å pixel^−1^. For the Backtracked Pol II-Rad26 sample, 9,167 movies were recorded in counting mode at a dose rate of 6.75 electrons pixel^−1^ s^−1^ with a total exposure time of 11 s sub-divided into 200-ms frames, for a total of 55 frames. The images were recorded at a nominal magnification of 36,000× resulting in an object-level pixel size of 1.16 Å pixel^−1^. For the Pol II(CPD)-Rad26 sample with Pol II lacking Rpb4/7, 955 movies were recorded in super-resolution mode at a dose rate of 5.34 electrons pixel^−1^ s^−1^ with a total exposure time of 13 s sub-divided into 250-ms frames, for a total of 44 frames. The images were recorded at a nominal magnification of 36,000× resulting in an object-level pixel size of 1.16 Å pixel^−1^ (0.58 Å per super-resolution pixel). For the Pol II(CPD)-Rad26-Elf1 sample, two datasets with total of 6,000 movies were recorded in counting mode at a dose rate of 6.9 electrons pixel^−1^ s^−1^ for the first dataset and 7.4 electrons pixel^−1^ s^−1^ for the second dataset with a total exposure time of 10 s sub-divided into 200-ms frames, for a total of 50 frames. The images were recorded at a nominal magnification of 36,000× resulting in an object-level pixel size of 1.16 Å pixel^−1^. See *SI Appendix*, Table S1 for details on cryo-EM data collection, refinement, and validation.

### Image Processing.

Movie frame alignment was performed using MotionCore2 (41) using the dose-weighted frame alignment option. CTF estimation was executed on the non-dose-weighted aligned micrographs using GCTF using the local defocus per particle option (42). Particle picking was performed using FindEM (43) with 2D averages selected from the initial processing serving as templates. Motion correction, CTF estimation, and particle picking were performed within the framework of Appion (44). Two-dimensional classification was performed to identify bad Pol II particles. Following 2D classification, an initial 3D classification was performed using a Pol II Elongation Complex model (PDB 1Y77) as reference. The 2D and initial 3D classifications were carried out using particles binned by 4 (4.64 Å pixel^−1^). The detailed processing schemes for each sample are shown in *SI Appendix*, Figs. S1, S3, S7, and S10. All initial refinements and classifications were done in Relion 3 (45). Once the final particles were selected, local and global ctf refinement were performed to further improve the resolution using cryoSPARC (46). The final map was refined in cryoSPARC using non-uniform refinement algorithm (47). The statistics for refinement of all maps are listed in *SI Appendix*, Table S1 and S2.

### Model Building.

For building the models of Pol II (CPD) Conformation 1 and 2, models of Pol II(CPD) complex (PDB accession 6O6C) (48) and Rpb4/7 of Pol II elongation complex model (PDB accession 5VVS) (8) were used as starting models for Pol II core (10 subunits) and Rpb4/7, respectively. The composite reference model of Pol II core and Rpb4/7 and the density maps were used as inputs in RosettaCM (49), in which 10 models were generated. A model with the best Rosetta energy was selected for each density map. Models were manually optimized in coot (50) and then refined using Rosetta Relax to further optimize the position and geometry of the amino acid side chains. The nucleic acids scaffold was manually built in coot. A selected model was refined using PHENIX real space refinement (51) with secondary structure restrains option followed by second round of Rosetta Relax, in which 10 models were generated. A model with the best score function was selected as the final model. The metals were manually added to each model followed by a final run of PHENIX real space refinement. The model of backtracked Pol II complex apo was built using the same steps described above except that the Pol II(CPD) complex Conformation 1 model was used as a starting model.

For building the model of Rad26 for Pol II(CPD)-Rad26, Backtracked Pol II-Rad26 complex, and Pol II(CPD)-Rad26 with Pol II lacking Rpb4/7, the model of Pol II-Rad26 stalled (PDB accession 5VVR) (8) was used as a reference. The Rad26 starting model was rigid body docked into the density map using UCSF Chimera (52). The N-terminal helix of Rad26 was manually adjusted or deleted in coot to best fit the density map. The composite reference model of optimized Rad26 and Pol II(CPD) Conformation 1 (built as described above) was used as a starting model in RosettaCM.

To build the model Pol II(CPD)-Rad26-Elf1, the reference models for Rad26 and Elf1 were selected based on homology detection using the hidden Markov model as implemented in HHpred (53). The segmented density of Rad26 and Elf1, and the reference models from HHpred were used as inputs to build their models using RosettaCM. Pol II was built using the composite models of Pol II 10 subunit (from PDB: 6O6C) and Rpb4/7 (from PDB: 5VVS) as described above. Nucleic acid scaffolds for all models were built in coot. The same steps described above were performed to improve position and geometry of the amino acids side chains. FSC curves of map-to-model were calculated in Rosetta. The validation statistics for all models are shown in *SI Appendix*, Table S1.

### Structure Analysis.

All figures were generated using UCSF ChimeraX (54). The cryo-EM maps were first segmented using Seggar (55) as implemented in UCSF Chimera. The segmented densities were colored in ChimeraX.

To generate difference maps for “Engaged” state minus “Open” state of Pol II(CPD)-Rad26, the cryo-EM maps were first low-pass filtered in SPIDER (56) using the FQ operation, with a “top-hat” function preserving frequencies below 0.1 (a resolution of 10 Å in our maps). The difference map was generated in ChimeraX with volume operation (vol) as follows: The filtered “Engaged” state was fitted into “Open” state map with “fitmap” command, the “Engaged” state was resampled on the grid of “Open” state map with “vol resample” command, and the “Open” state map was subtracted from the resampled “Engaged” state map with “vol subtract” command. The same steps were followed to generate difference map for Pol II(CPD)-Rad26 (“Engaged” state) minus Backtracked Pol II-Rad26 and Pol II(CPD)-Rad26-Elf1 minus Pol II(CPD)-Rad26.

The consensus refinement and the masks used in multi-body refinement were prepared in Relion 3 using the default options. Multi-body refinement generated 10 structures, which describe flexibility along each eigenvector. To visualize flexibility along eigenvector 1 and 2 for Pol II(CPD)-Rad26 (“Engaged” state), the model of Rpb4/7 was segmented out from Pol II(CPD)-Rad26 “Engaged” state model and was rigid-body fitted separately into each one of the 10 structures from multi-body refinement. The models were fitted using fitmap command in ChimeraX. The same steps were followed to visualize the flexibility along eigenvector 1 and 2 for Pol II(CPD) but using the consensus refinement of Pol II(CPD) to generate masks and as an input for multi-body refinement. The segmented model of Rpb4/7 chains from Pol II(CPD) (lacking Rad26) model was used for rigid-body fitting.

To obtain the cross-correlation coefficients between the Rpb4/7 model and the different cryo-EM maps shown in *SI Appendix*, Fig. S5, the cryo-EM maps for the “Engaged” and “Open” states of Pol II(CPD)-Rad26 were aligned with the fitmap function in ChimeraX. Then, the full complex model was aligned to its corresponding cryo-EM density. To calculate cross-correlation coefficients, the model of Rpb4/7 from Pol II(CPD)-Rad26 (“Engaged” state) was fitted into the segmented Rpb4/7 density from “Open” and “Engaged” states while disabling the options for allowing any rotations and shifts. The same steps were performed to calculate the cross-correlation for the fitting of Rpb4/7 model for the “Engaged” state of Pol II(CPD)-Rad26 into the map of Backtracked Pol II-Rad26 (*SI Appendix*, Fig. S6).

### Yeast Strain Construction.

Yeast strains used in this study are listed in *SI Appendix*, Table S2. Plasmids expressing 6×FLAG tagged wild-type (p6FRAD26) and indicated mutant Rad26 proteins were created using the pRS415 vector (57). Yeast strains expressing 6×FLAG tagged wild-type and mutant Rad26 proteins were created by transforming the yeast strain CR18 (58) with the plasmids.

To make *elf1-*Δ*C* mutant strain, a *URA3* fragment from pRS306 was PCR amplified with Primer 1 and Primer 2 (see below) to replace the part of *ELF1* open reading frame that encodes C-terminal 60 amino acids (with the incorporation of a TAA stop codon immediately after the amino acid 58). The PCR-cassette was transformed into cells using the method described previously (59). The resulting mutant strains were further confirmed by sequencing. Primers are listed below:

Primer 1:

T​GAT​GTA​TAT​AGT​GAT​TGG​TTT​GAC​GCC​GTC​GAA​GAA​GTC​AAT​TCT​GGC​CGT​GGA​TAA​CCTGATGCGGTATTTTCTCC.

Primer 2:

T​TAA​AAT​ATA​AAA​TAT​ATA​TGA​CCT​AAG​TAA​ATA​TGG​TTT​TTT​CTC​AGG​ACC​GGA​CGGCATCAGAGCAGATTGTA

All genotypes of yeast strains are listed in *SI Appendix*, Table S2.

### Mapping Repair of UV-Induced CPDs.

Yeast cells were cultured at 30 °C to late log phase (OD600 ≈ 1.0), irradiated with 120 J/m2 of ~254 nm UV and incubated at 30 °C. At different timepoints of the post-UV incubation, aliquots were taken, and the genomic DNA was isolated. The CPDs remaining in the AGP2, RPB2, and YEF3 genes in the isolated genomic DNA were analyzed using the Lesion-Adjoining Fragment Sequencing (LAF-Seq) method (60). Sequencing reads whose 3′ ends adjoin the sites of CPDs remained in the genomic DNA were aligned to the TS and/or NTS sequences of the AGP2, RPB2, and YEF3 gene fragments. Reads corresponding to CPDs at individual sites along the gene fragments were counted after subtraction of the background counts (in the unirradiated samples) by using codes in R. To better illustrate the CPD induction and repair profiles, pseudo images whose band intensities correspond to the counts of aligned sequencing reads were generated using codes in R.

### UV Survival Assay.

Yeast cells were grown at 30 °C to optical density (OD) of 3 at 600 nm and diluted to OD 0.6 in YPD medium. Cells were plotted on YPD plate with fivefold (Fig. 2) or 10-fold (Fig. 5) serial dilutions. Once dried, the plates were UV irradiated with UV crosslinker (FisherBiotech® FB-UVXL-1000) in dark room and wrapped in foil after irradiation. Plates were incubated in the dark for 2 to 5 d at 30 °C before imaging.

## Supplementary Material

Appendix 01 (PDF)

## Data Availability

Cryo-EM maps and corresponding models have been deposited in the EM Data Bank and Protein Data Bank, respectively. Accession codes can be found in *SI Appendix*, Table S1. Pol II(CPD)-Rad26 “Engaged”: 8TUG and 41623; Pol II(CPD)-Rad26 “Open”: 8TVP and 41647; Pol II(CPD) (conformation 1): 8TVW and 41653; Pol II(CPD) (conformation 2): 8TVX and 41654; Pol II(CPD)-Rad26 (10-subunit): 8TVQ and 41648; Pol II-Rad26 (Backtracked): 8TVS and 41650; Pol II (Backtracked): 8TVV and 41652; and Pol II(CPD)Rad26-Elf1 (“Closed” state): 8TVY and 41655.
