# Supplementary material for: Elf1 promotes Rad26’s interaction with lesion-arrested Pol II for transcription-coupled repair
Source: Proc Natl Acad Sci U S A. 2024 Jan 9;121(3):e2314245121. doi: 10.1073/pnas.2314245121 (PMC10801861; doi:10.1073/pnas.2314245121)
Supplement: Supplementary file 1 — Appendix 01 (PDF) [file pnas.2314245121.sapp.pdf]

Supplementary Information for

ELF1 promotes Rad26's interaction with lesion-arrested Pol II for transcription-coupled repair

Reta D Sarsam[1,6†], Jun Xu[2,7,†], Indrajit Lahiri[1,8], Wenzhi Gong[5], Qingrong Li[2], Juntaek Oh[2,9], Zhen Zhou[3], Peini Hou[2], Jenny Chong[2], Nan Hao[3], Shisheng Li[5,*], Dong Wang[1,2,4*], and Andres E. Leschziner[1,3*]

[1] Department of Cellular & Molecular Medicine, University of California San Diego, La Jolla, CA 92093
[2] Division of Pharmaceutical Sciences, Skaggs School of Pharmacy & Pharmaceutical Sciences, University of California San Diego, La Jolla, CA 92093
[3] Department of Molecular Biology, School of Biological Sciences, University of California San Diego, La Jolla, CA 92093
[4] Department of Chemistry and Biochemistry, University of California San Diego, La Jolla, CA 92093
[5] Department of Comparative Biomedical Sciences, School of Veterinary Medicine, Louisiana State University, Baton Rouge, LA 70803
[6] Present Address: Charles River Laboratories, Durham, NC 27703
[7] Present Address: Genetics and Metabolism Department, The Children's Hospital, School of Medicine, Zhejiang University, National Clinical Research Center for Child Health, Hangzhou 310052, China.
[8] Present Address: School of Biosciences, University of Sheffield, UK
[9] Present Address: Department of Pharmacy, College of Pharmacy, Kyung Hee University; Seoul 02447, Republic of Korea

[†] These authors contributed equally to this work.
* Corresponding authors: dongwang@ucsd.edu, shli@lsu.edu, aleschziner@ucsd.edu

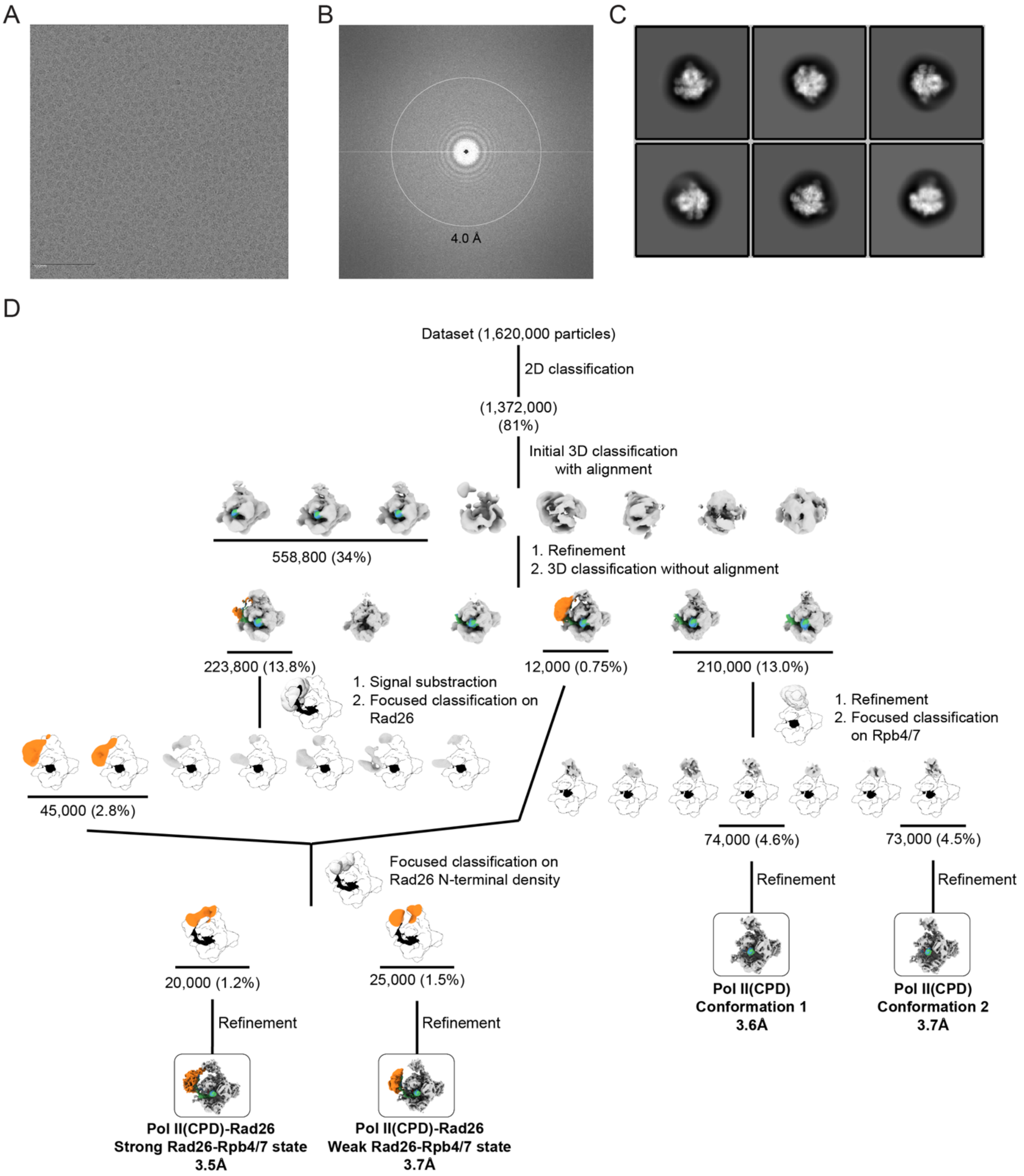

Figure S1. Cryo-EM structure determination of the Pol II(CPD)-Rad26 and Pol II(CPD) complexes. (A-C) Representative micrograph **(A),** power spectrum **(B),** and representative 2D class averages **(C)** of Pol II(CPD)-Rad26 complexes. **(D)** Schematic of the strategy used to sort out the dataset into Pol II(CPD)-Rad26 "Engaged" and "Open" Rad26-Rpb4/7 states, and Pol II(CPD) conformations 1 and 2. Focused 3D classification was performed without alignment unless otherwise noted. The number of particles contributing to each selected structure is indicated. The percentages shown are related to the total number of particles picked from the micrographs. The indicated resolution corresponds to the 0.143 Fourier shell correlation (FSC) based on gold-standard FSC curves (see Figure S2).

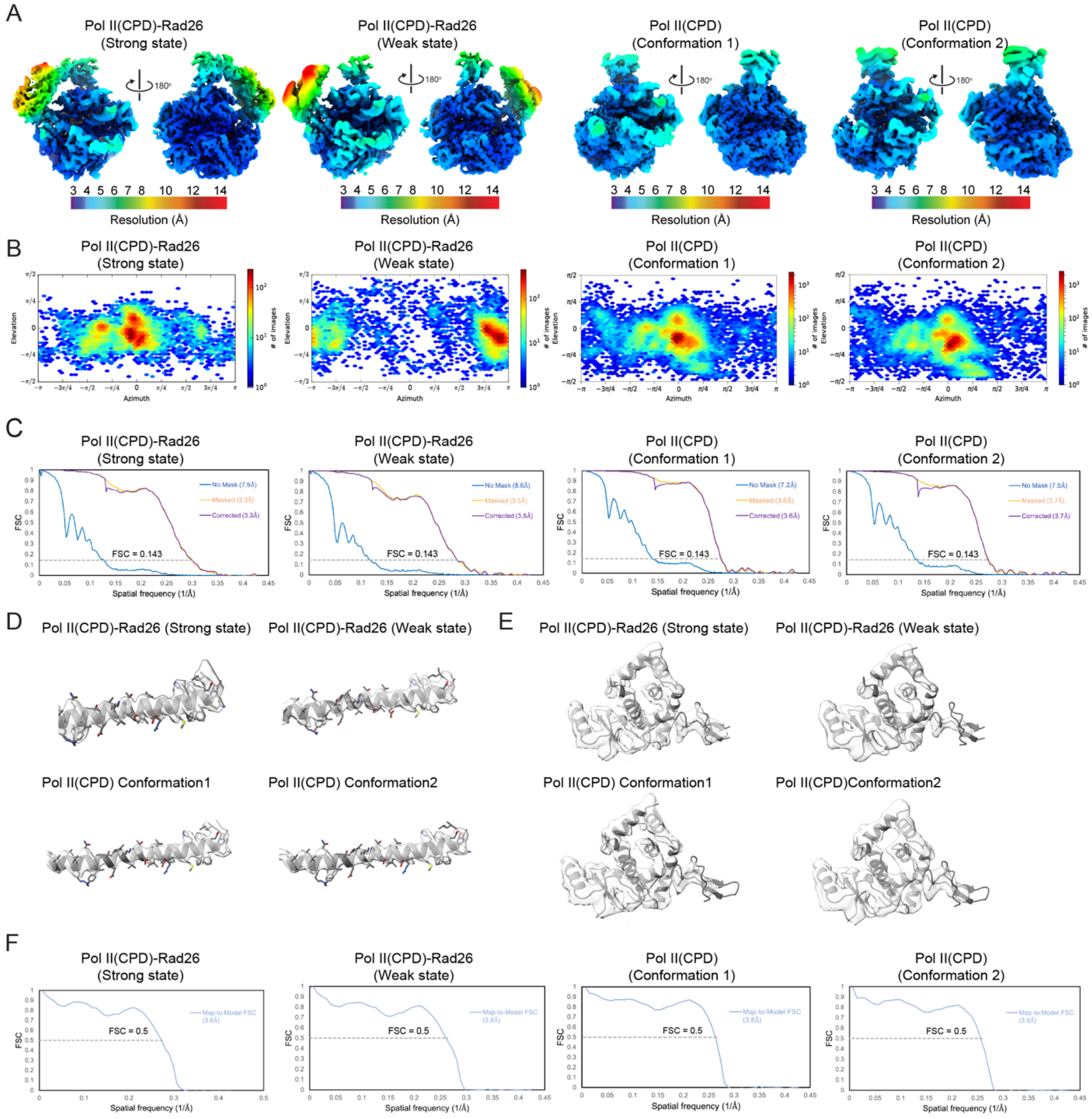

Figure S2. Analysis of the Pol II(CPD)-Rad26 and Pol II(CPD) cryo-EM maps. (A) Front and back views of locally filtered maps, colored by local resolution, of Pol II(CPD)-Rad26 "Engaged" and "Open" states, and Pol II(CPD) conformations 1 and 2. **(B, C)** Euler angle distribution of particle images **(B)** and FSC plots **(C)** for the maps shown in (A). **(D-E)** Close-ups of the cryo-EM densities corresponding to the Rpb1 Bridge helix **(D)**, and the Rpb2/Rpb9 'Jaw' of Pol II **(E)** for the indicated structures with the models fitted in. **(F)** FSC curves for map-to-model fits for the maps shown in (A). The 0.5 FSC line is shown.

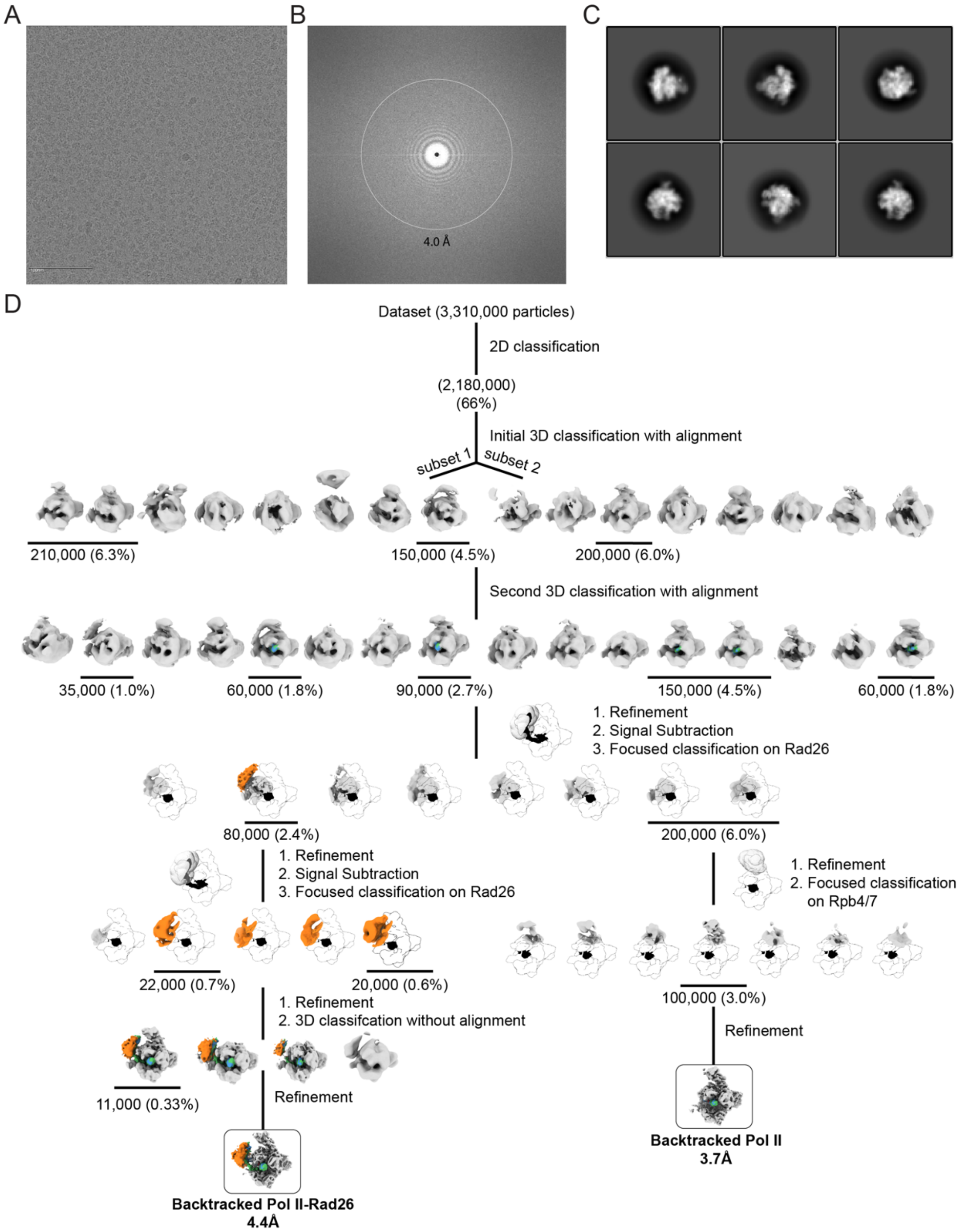

Figure S3. Cryo-EM structure determination of the Backtracked Pol II-Rad26 and Backtracked Pol II complexes. (A-C) Representative micrograph **(A),** Power spectrum **(B),** and representative 2D class averages **(C)** of Backtracked Pol II-Rad26 complexes. **(D)** Schematic representation of the strategy used to sort out the dataset into Backtracked Pol II-Rad26 and Backtracked Pol II. Focused 3D classification was performed without alignment unless otherwise noted. The number of particles contributing to each selected structure is indicated. The percentages shown are related to the total number of particles picked from micrographs. The indicated resolution corresponds to the 0.143 Fourier shell correlation (FSC) based on gold-standard FSC curves (see Figure S4).

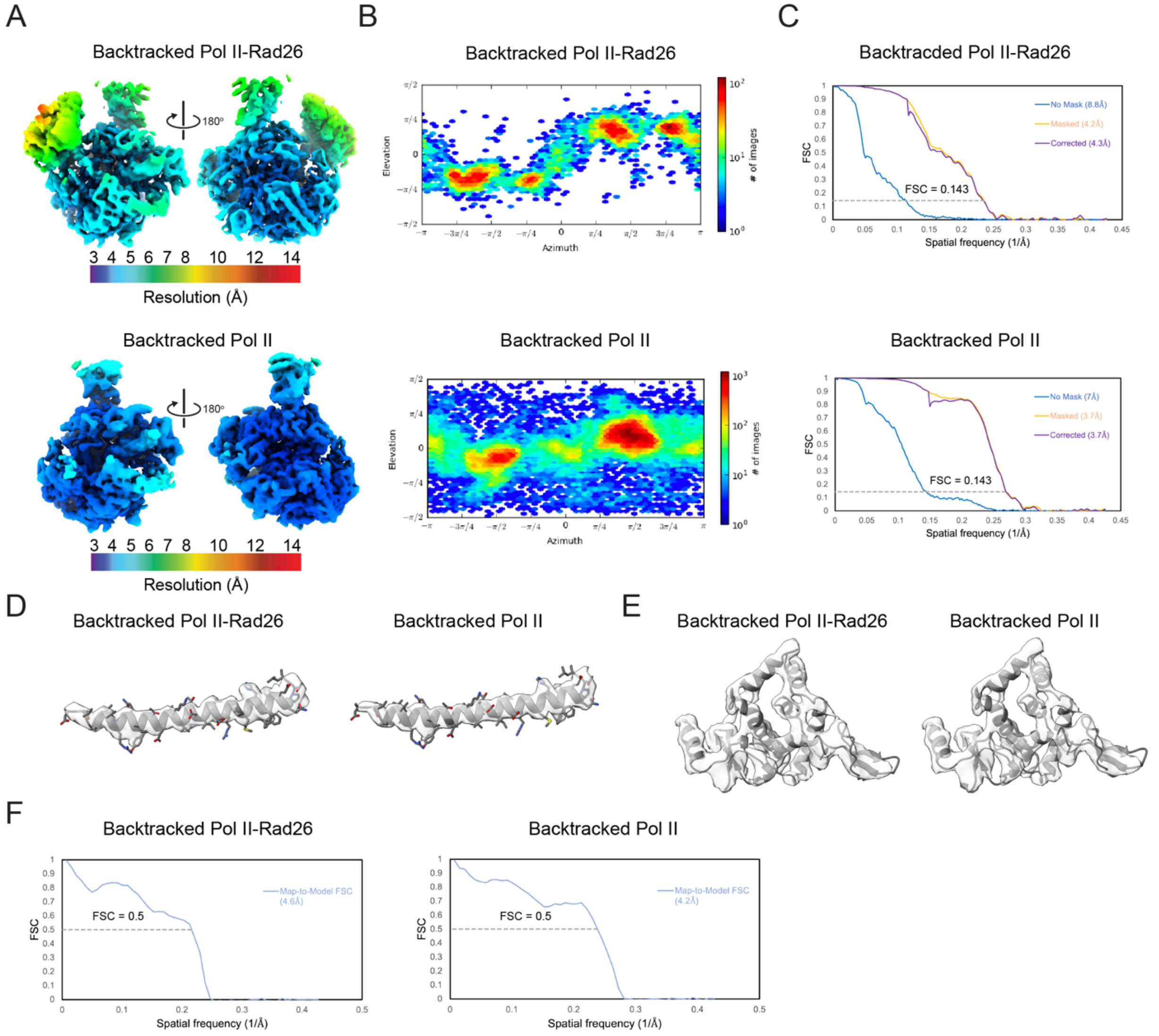

Figure S4. Analysis of the Backtracked Pol II-Rad26 and Backtracked Pol II cryo-EM maps. (A) Front and back views of locally filtered maps, colored by local resolution, of Backtracked Pol II-Rad26 and Backtracked Pol II. **(B, C)** Euler angle distribution of particle images **(B)** and FSC plots **(C)** for the maps shown in (A). **(D-E)** Close-ups of the cryo-EM densities corresponding to the Rpb1 Bridge helix **(D)**, and the Rpb2/Rpb9 'Jaw' of Pol II **(E)** for the indicated structures with the models fitted in. **(F)** FSC curves for map-to-model fits for the maps shown in (A). The 0.5 FSC line is shown.

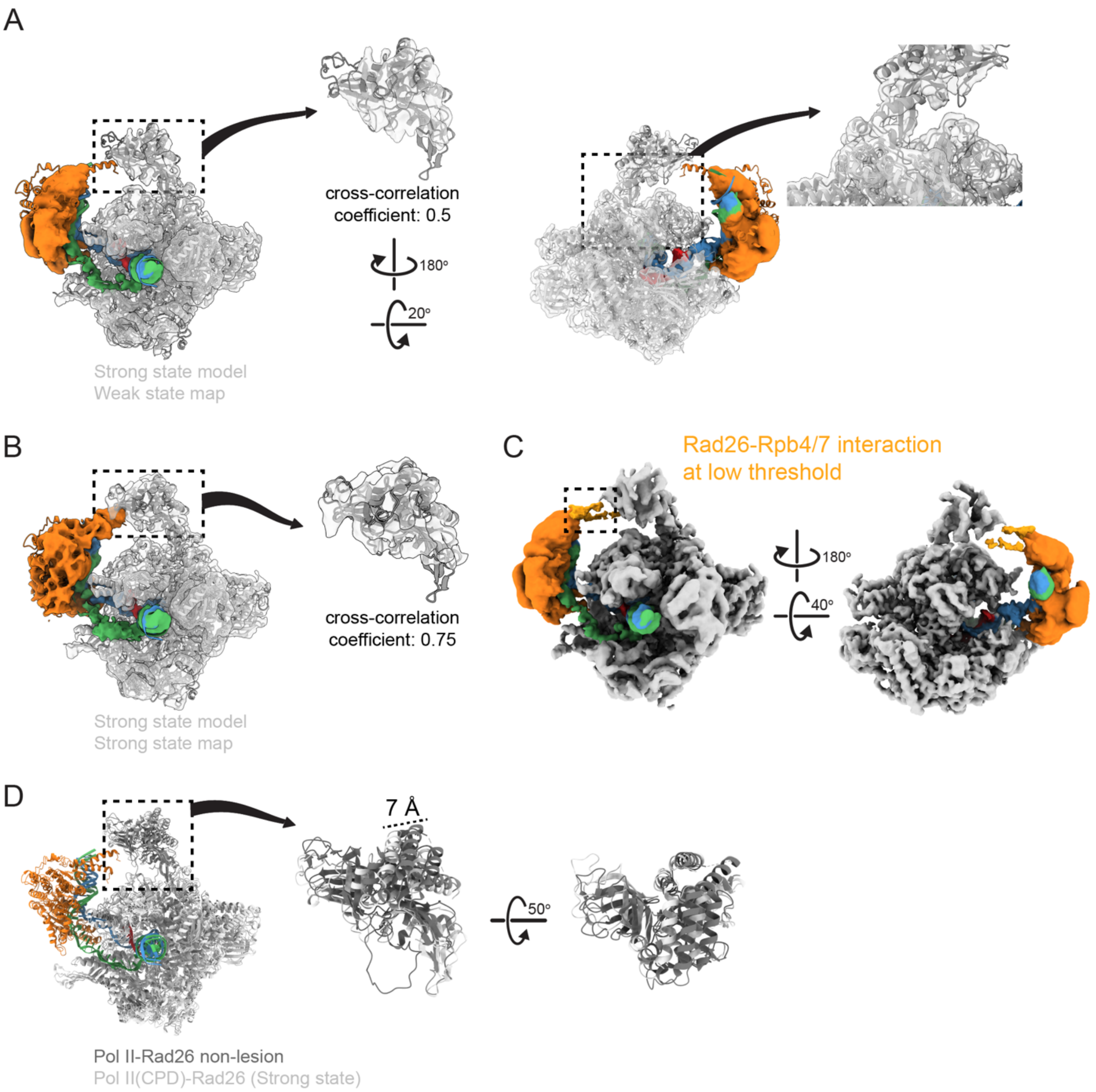

Figure S5. Structural analysis of Pol II-Rad26 and Pol II(CPD)-Rad26 complexes. (A) Two views are shown of the model for the “Engaged” Rad26-Rpb4/7 state of Pol II(CPD)-Rad26 fitted into the cryo-EM map of the “Open” Rad26-Rpb4/7 state of Pol II(CPD)-Rad26, with zoomed-in view of the cryo-EM density of Rpb4/7 with the model fitted in shown to their right. Fitting of the model into the map was driven by the core of Pol II. The cross-correlation coefficient for the fitting of the Rpb4/7 model for the “Engaged” state into the map of the “Open” state was 0.5 as reported by Fit-in-Map in ChimeraX. **(B)** Model for the “Engaged” Rad26-Rpb4/7 state fitted into the cryo-EM map for the same state. The cross-correlation coefficient for the fitting of the Rpb4/7 model for the “Engaged” state into the map of the “Engaged” state was 0.75 as reported by Fit-in-Map in ChimeraX. **(C)** Cryo-EM map of the “Open” Rad26-Rpb4/7 state of Pol II(CPD)-Rad26 shown at lower threshold, where the interaction between Rad26 and Rpb4/7 becomes apparent. **(D)** Superposition of models for Pol II(CPD)-Rad26 (“Engaged” state) and Pol II-Rad26 (no lesion).

The models were aligned using the core of Pol II. Two zoomed-in views of Rpb4/7 from the two models are shown to the right.

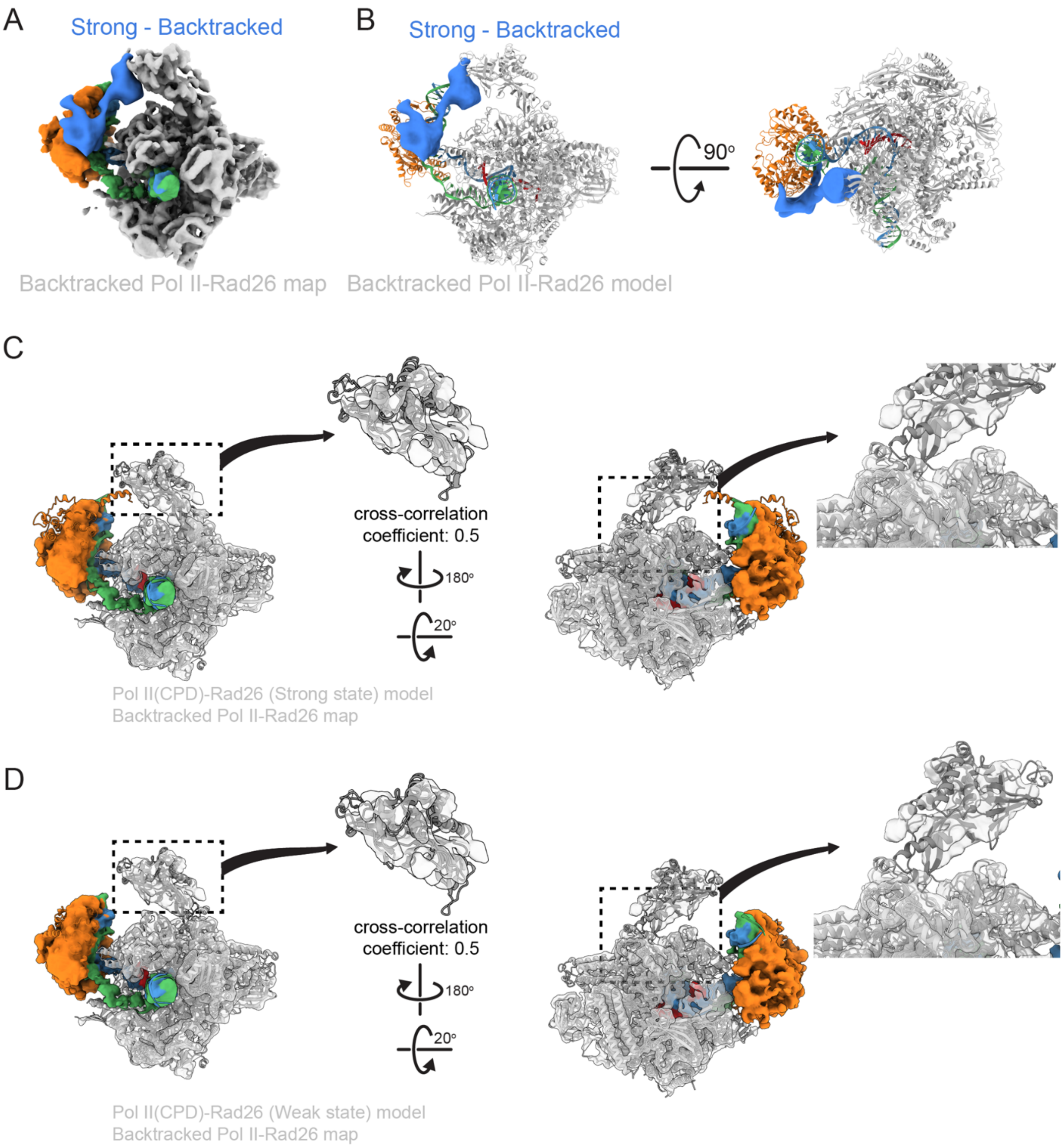

Figure S6. The Rad26-Rpb4/7 interaction is weakest in Backtracked Pol II-Rad26. (A, B) Difference map (in blue) calculated by subtracting Backtracked Pol II-Rad26 from Pol II(CPD)-Rad26 ("Engaged" state), displayed on either **(A)** the cryo-EM density or **(B)** the atomic model for Backtracked Pol II-Rad26. **(C)** Two views are shown of the model for Pol II(CPD)-Rad26 ("Engaged" state) fitted into the cryo-EM map of the Backtracked Pol II-Rad26, with zoomed-in views of the cryo-EM density of Rpb4/7 with the model fitted in shown to their right. The cross-

correlation coefficient for the fitting of the Rpb4/7 model for the "Engaged" state into the map of Backtracked Pol II-Rad26 was 0.5 as reported by Fit-in-Map in ChimeraX. **(D)** same as (C), but with the model for Pol II(CPD)-Rad26 ("Open" state) fitted into the cryo-EM map of the Backtracked Pol II-Rad26. The cross-correlation coefficient for the fitting of the Rpb4/7 model for the "Open" state into the map of Backtracked Pol II-Rad26 was 0.5 as reported by Fit-in-Map in ChimeraX.

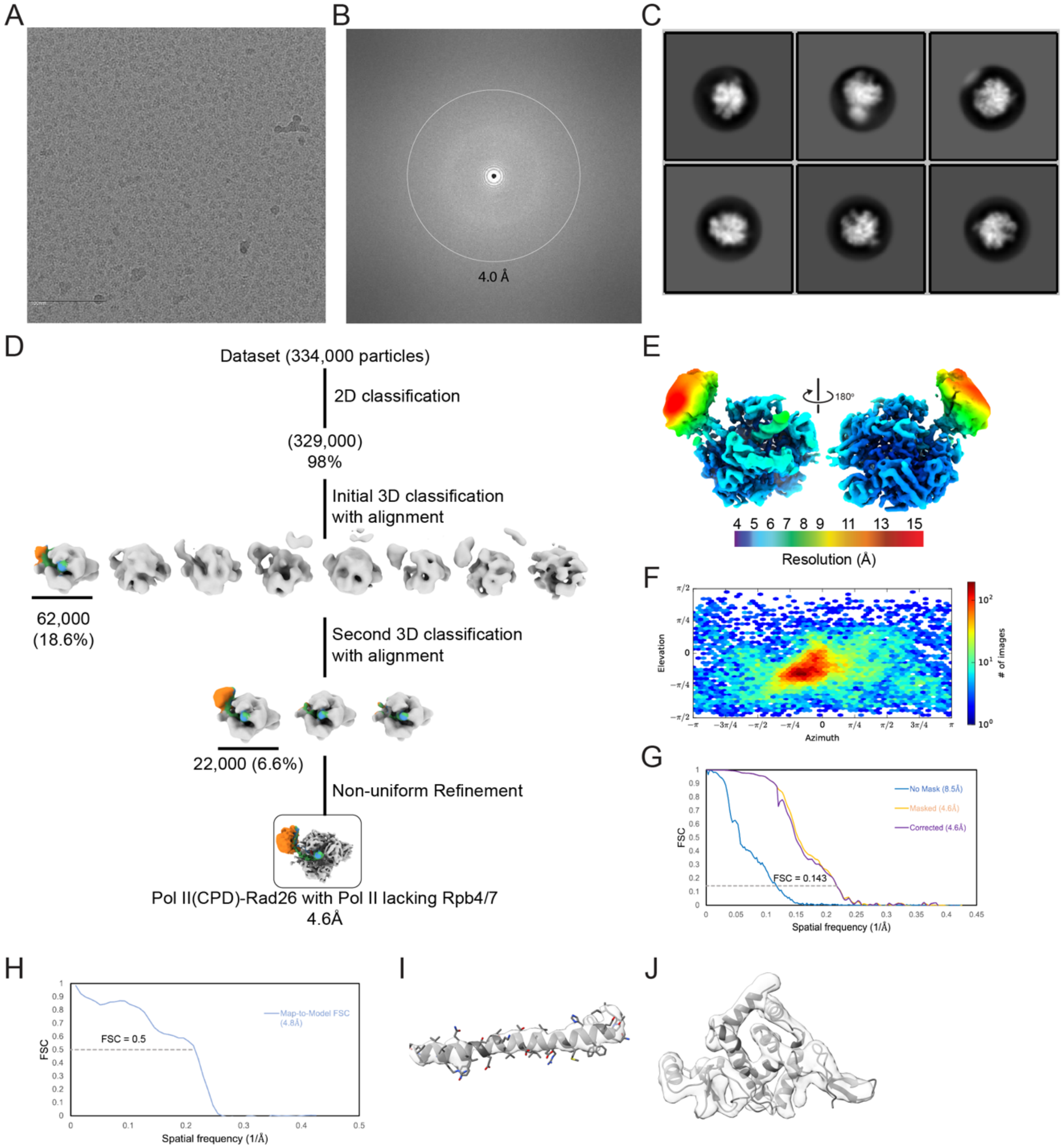

Figure S7. Cryo-EM structure determination and analysis of the Pol II(CPD)-Rad26 complex with Pol II lacking Rpb4/7. (A-C) Representative micrograph **(A)**, power spectrum **(B),** and representative 2D class averages **(C)** of Pol II(CPD)-Rad26 with Pol II lacking Rpb4/7. **(D)** Schematic representation of the strategy used to sort out the complex particles. The number of

particles contributing to each selected structure is indicated. The percentages shown are related to the total number of particles picked from micrographs. The indicated resolution corresponds to the 0.143 Fourier shell correlation (FSC) based on gold-standard FSC curves. **(E)** Front and back views of locally filtered maps, colored by local resolution, of Pol II(CPD)-Rad26 with Pol II lacking Rpb4/7. **(F-H)** Euler angle distribution of particle images **(F)**, FSC plot **(G)** and FSC curve for the map-to-model fit **(H)** for the map shown in (E). **(I,J)** Close-ups of the cryo-EM densities corresponding to the Rpb1 Bridge helix **(I)**, and the Rpb2/Rpb9 'Jaw' of Pol II **(J)** for the indicated structure with the model fitted in

.

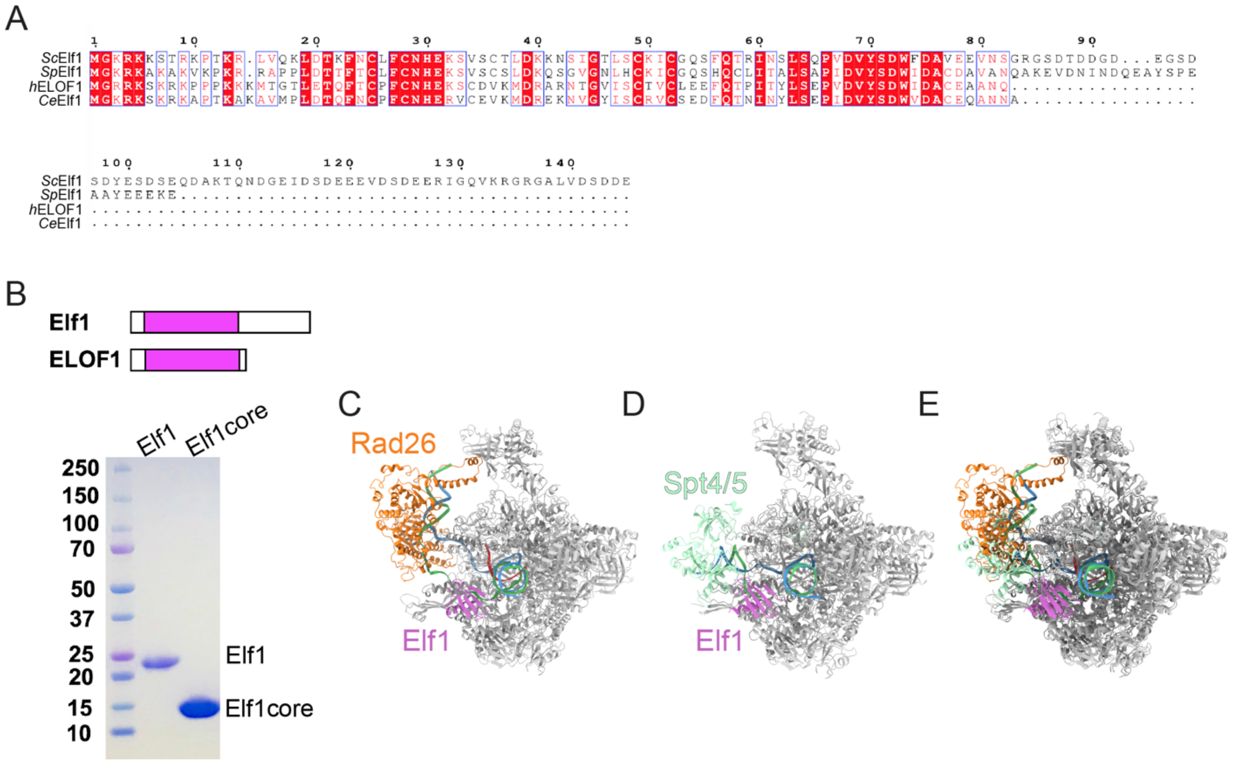

Figure S8. Purification of Elf1 and comparison of our structure of Pol II(CPD)-Rad26-Elf1 with the published structure of Pol II-Spt4/5-Elf1 (A) Sequence alignment of Elf1 orthologs from *S.cerevisiae* (*Sc*), *S.pombe* (*Sp*), humans (*h*) and *C. elegans* (*Ce*). **(B)** SDS-PAGE of purified Elf1 and Elf1core, shown schematically at the top. **(C-D)** Structures of **(C)** Pol II(CPD)-Rad26-Elf1 (this work) and **(D)** Pol II-Spt4/5-Elf1 (PDB: 6J4Y)(30). **(E)** Superimposition of the two models in (C) and (D).

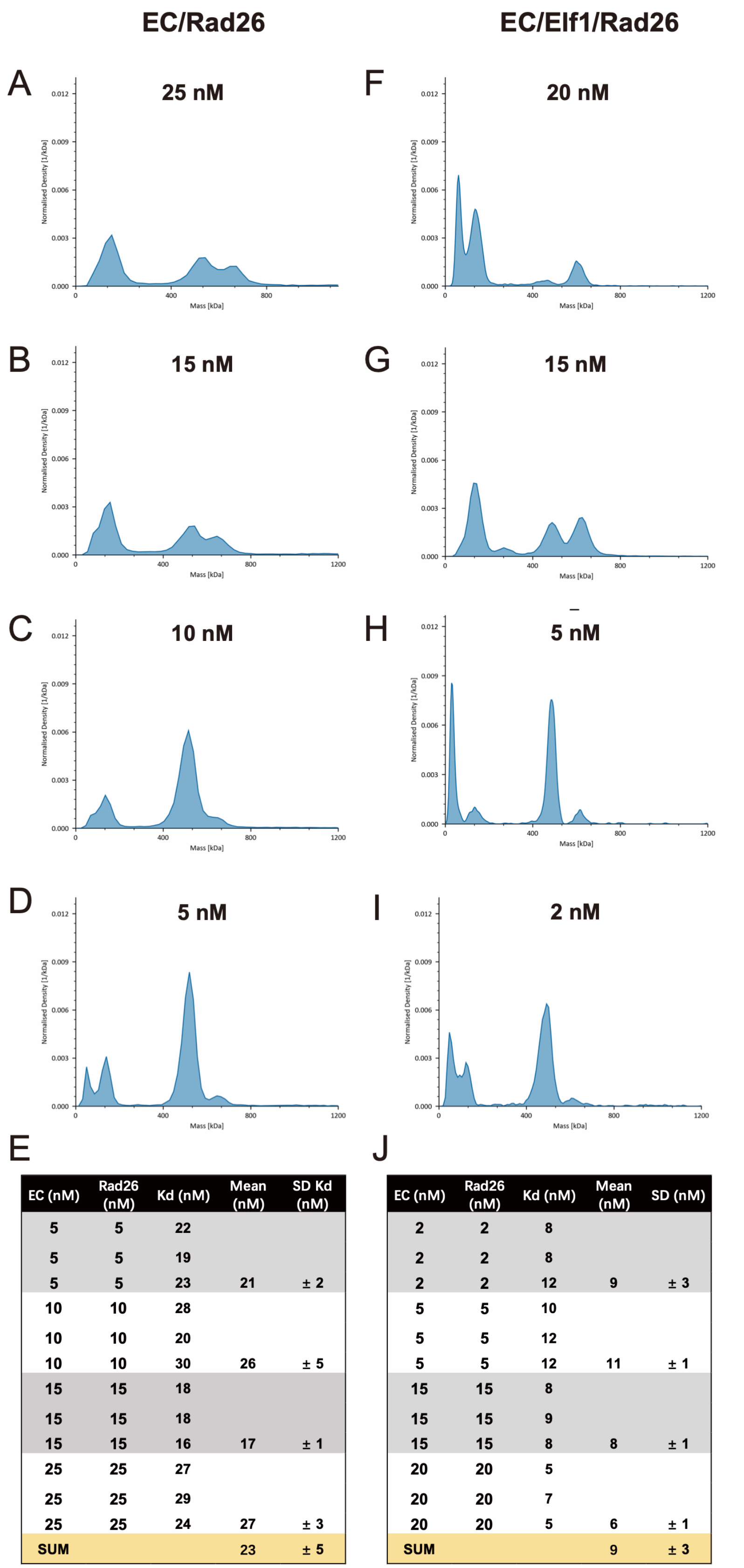

E

EC (nM)	Rad26 (nM)	Kd (nM)	Mean (nM)	SD Kd (nM)
5	5	22		
5	5	19		
5	5	23	21	± 2
10	10	28		
10	10	20		
10	10	30	26	± 5
15	15	18		
15	15	18		
15	15	16	17	± 1
25	25	27		
25	25	29		
25	25	24	27	± 3
SUM			23	± 5

J

EC (nM)	Rad26 (nM)	Kd (nM)	Mean (nM)	SD (nM)
2	2	8		
2	2	8		
2	2	12	9	± 3
5	5	10		
5	5	12		
5	5	12	11	± 1
15	15	8		
15	15	9		
15	15	8	8	± 1
20	20	5		
20	20	7		
20	20	5	6	± 1
SUM			9	± 3

Figure S9. Elf1 enhances the binding of Rad26 to CPD-lesion arrested Pol II complex. (A-D) Representative mass photometry plots for measurement of Rad26 binding with Pol II (EC) stalled at a CPD lesion in the absence of Elf1 (measured at 25 nM, 15 nM, 10 nM, and 5 nM of final concentration of 1:1 Rad26 and Pol II EC, respectively). **(E)** K_d values for the interaction between Pol II (EC) stalled at a CPD lesion and Rad26 in the absence of Elf1 were determined from multiple repeats of the experiment in (A-D). **(F-I)** Representative mass photometry plots for measurement of Rad26 binding with Pol II (EC) stalled at a CPD lesion in the presence of 500 nM Elf1 (measured at 20 nM, 15 nM, 5 nM, and 2 nM of final concentration of 1:1 Rad26 and Pol II EC-Elf1, respectively)**. (J)** K_d values for the interaction between Pol II (EC) stalled at a CPD lesion and Rad26 in the presence of Elf1 were determined from multiple repeats of the experiment in (F-I).

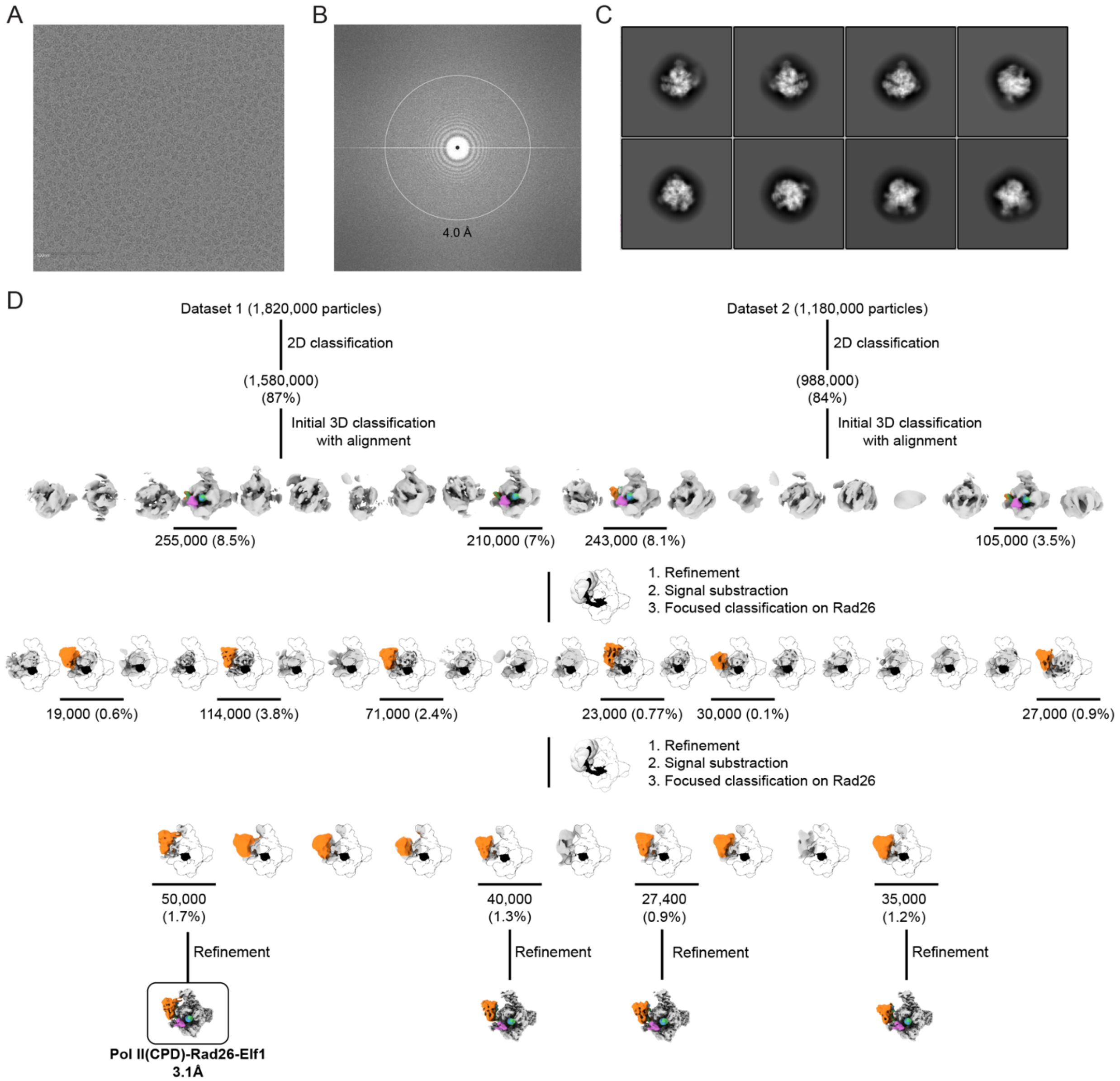

Figure S10. Cryo-EM structure determination of the Pol II(CPD)-Rad26-Elf1 complex. (A-C) Representative micrograph **(A),** power spectrum **(B),** and representative 2D class averages **(C)** of the Pol II(CPD)-Rad26-Elf1 complex. **(D)** Schematic of the strategy used to sort out the dataset. Focused 3D classification was performed without alignment unless otherwise noted. The number of particles contributing to each selected structure is indicated. The percentages shown are related to the total number of particles picked from the micrographs. The indicated resolution corresponds to the 0.143 Fourier shell correlation (FSC) based on gold-standard FSC curves (see Figure S11).

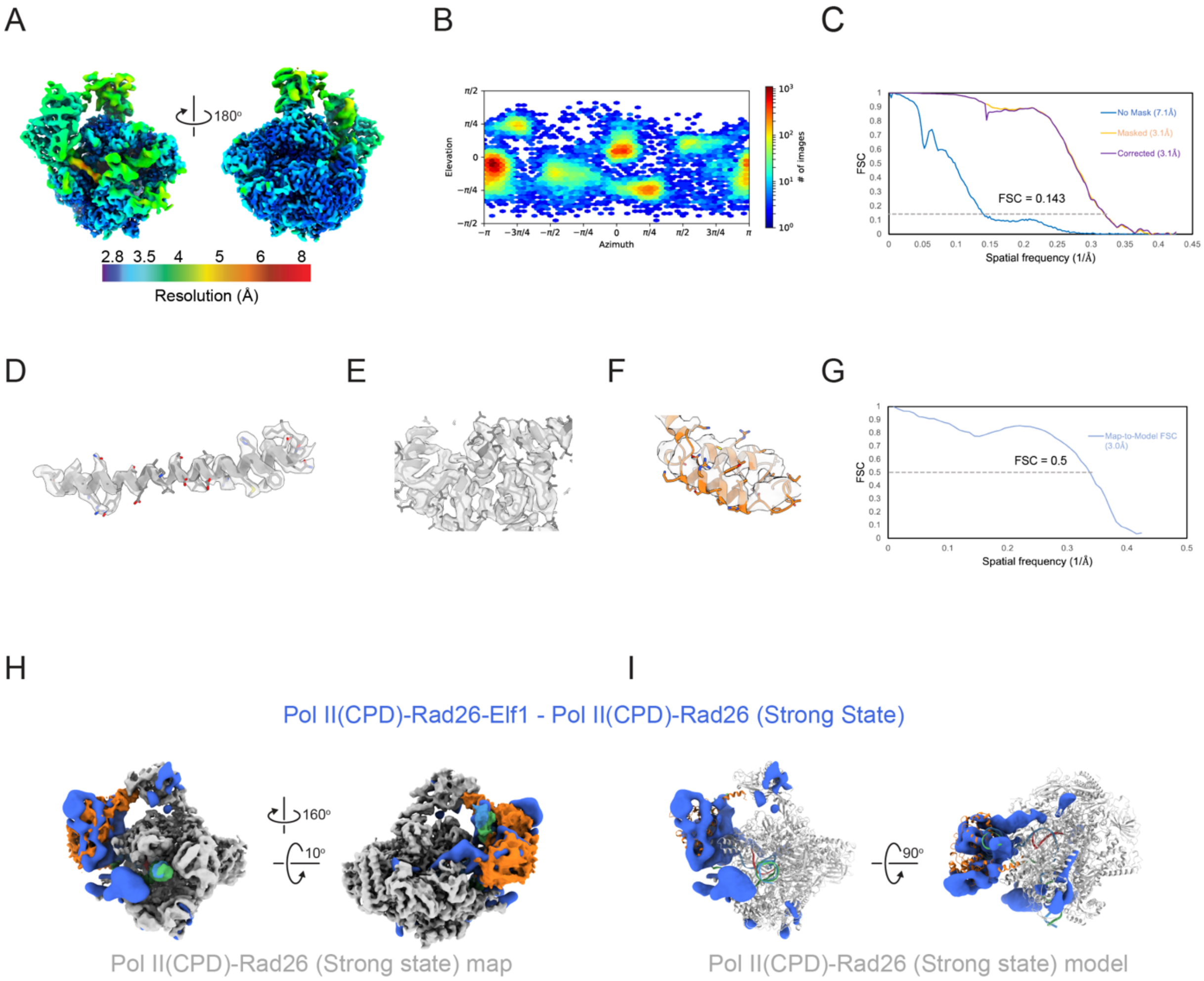

Figure S11. Analysis of the Pol II(CPD)Rad26-Elf1 cryo-EM map. (A) Front and back views of locally filtered maps, colored by local resolution. **(B, C)** Euler angle distribution of particle images **(B)** and FSC plots **(C)** for the map shown in (A). **(D-F)** Close-ups of the cryo-EM densities corresponding to the Rpb1 Bridge helix **(D)**, the Rpb2/Rpb9 'Jaw' of Pol II **(E)** and the Rad26 HD2-1 'wedge' **(F)** for the indicated structures with the models fitted in. **(G)** FSC curves for map-to-model fit for the map shown in (A). The 0.5 FSC line is shown. **(H, I)** Difference map (in blue) calculated by subtracting Pol II(CPD)Rad26 ("Engaged" state) from Pol II(CPD)-Rad26-Elf1, displayed on either **(H)** the cryo-EM density or **(I)** the atomic model for Pol II-CDP-Rad26 ("Engaged" state).

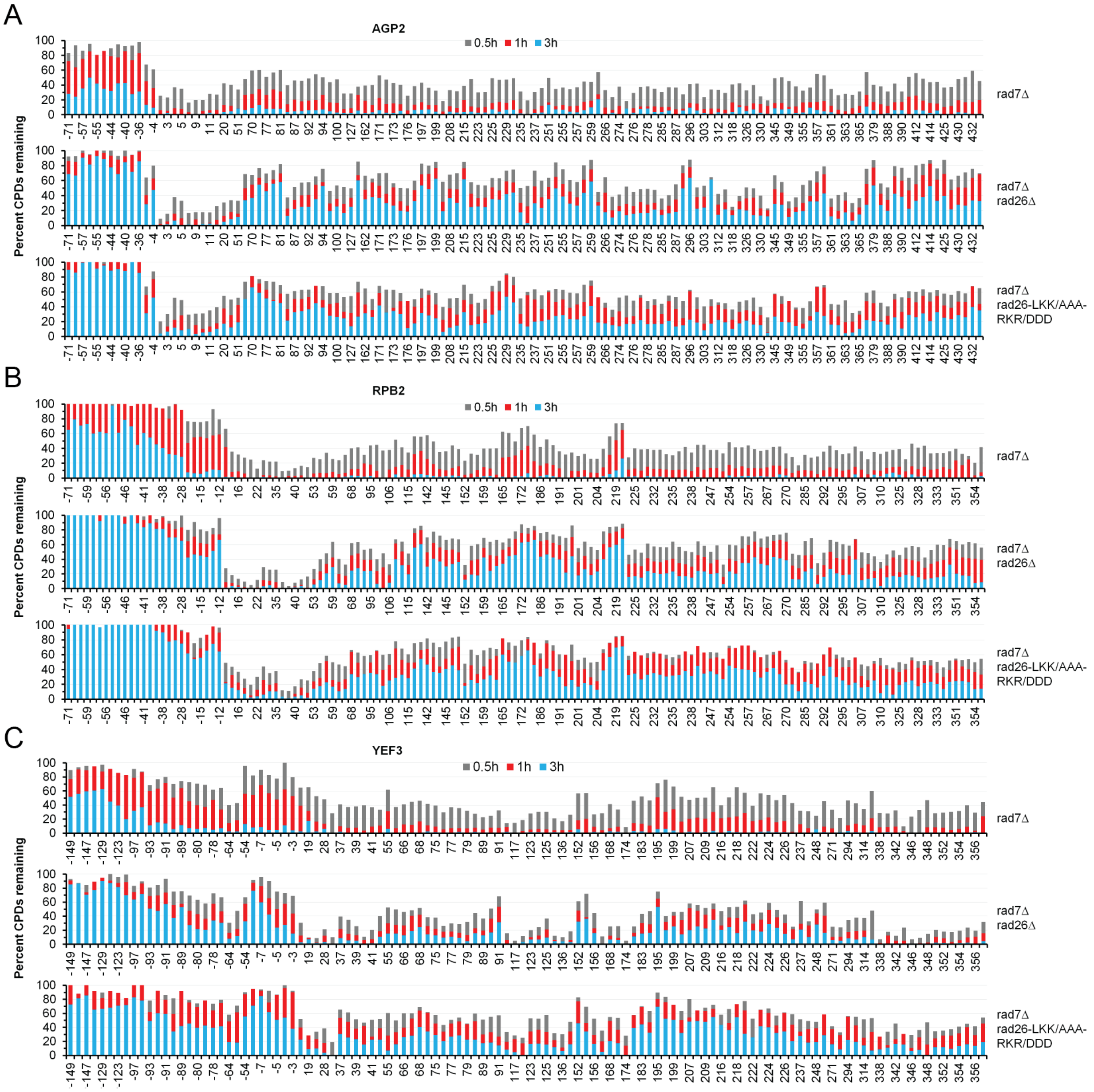

Figure S12 Base-resolution measurement of remaining CPD distributions at different loci after TC-NER. (A-C) Fraction (%) of CPDs at the indicated times of repair incubation along the AGP2 **(A)**, RPB2 **(B)** and YEF3 **(C)** loci of the indicated strains. Numbers at the bottom of each plot indicating the nucleotide positions of the loci are relative to the major TSS (+1).

Table S1. Cryo-EM data collection, refinement and validation statistics

	Pol II(CPD)-Rad26 Dataset				10-subunit
	"Engaged" state	"Open" state	Pol II(CPD) Conf1	Pol II(CPD) Conf2	
**PDB**	8TUG	8TVP	8TVW	8TVX	8TVQ
**EMDB**	41623	41647	41653	41654	41648
**Data Collection**					
Microscope			Talos Arctica		Talos Arctica
Camera			K2 Summit		K2 Summit
Camera Mode			Counting		Super-Res
Voltage (kV)			200		200
Magnification			36,000		36,000
Pixel Size (Å/pixel)			1.16		1.16
Dose rate (e-/Å^2 second)			8.4		4
Total dose (e-/Å^2)			59		52
Number of frames			47		52
Defocus range (µm)			0.6-2.5		0.6-2.5
Micrographs collected (no.)			3,358		955
Initial particle (no.)			1,620,000		334,000
Final particle (no.)	20,000	25,000	74,000	73,000	22,000
**Refinement**					
Initial model used			1Y77		1Y77
Final resolution (Å) (0.143 FSC threshold)	3.5	3.7	3.6	3.7	4.6
Map sharpening *B* factor (Å^2)	-73	-68	-87	-101	-153
**Model Refinement**					
Map-to-model resolution (Å) (0.5 FSC threshold)	3.6	3.8	3.8	3.9	4.8
Model Composition					
Nonhyrogen atoms	71,184	69,002	61,459	60,948	64,987
Protein residues	4,251	4,182	3,748	3,748	3,869
Nucleotides	103	103	56	56	100
Ligands	9	9	9	9	9
B factor (Å^2)	244	205	123	170	283
R.m.s. deviations					
Bond length (Å)	0.003	0.003	0.003	0.003	0.003
Bond angle (°)	0.56	0.54	0.59	0.60	0.644
Validation					
MolProbity score	1.7	1.8	1.7	1.7	2.1
Clash score	6.7	8.6	7.0	9.2	14.9
Poor rotamers (%)	0	0	0	0	0
Ramachandran					
Favored (%)	95.53	95.60	96.0	95.82	93.79
Allowed (%)	4.4	4.3	4.0	4.1	6.1
Disfavored (%)	0.07	0.1	0.05	0.08	0.11

Table S1. Cryo-EM data collection, refinement and validation statistics (continued)

	Backtracked Pol II-Rad26 Dataset		Pol II(CPD) Rad26-Elf1
	**Backtracked Pol II-Rad26**	**Backtracked Pol II**	**("Closed" state)**
**PDB**	8TVS	8TVV	8TVY
**EMDB**	41650	41652	41655
**Data Collection**			
Microscope	Talos Arctica		Talos Arctica
Camera	K2 Summit		K2 Summit
Camera Mode	Counting		Counting
Voltage (kV)	200		200
Magnification	36,000		36,000
Pixel Size (Å/pixel)	1.16		1.16
Dose rate (e-/Å^2 second)	5		5/5.5
Total dose (e-/Å^2)	55		50/55
Number of frames	55		50
Defocus range (µm)	0.6-2.5		0.6-2.5
Micrographs collected (no.)	9,167		8,000
Initial particle (no.)	3,310,000		3,000,000
Final particle (no.)	11,000	100,000	50,000
**Refinement**			
Initial model used	1Y77	1Y77	1Y77
Final resolution (Å) (0.143 FSC threshold)	4.4	3.7	3.1
Map sharpening *B* factor (Å^2)	-92	-117	-85
**Model Refinement**			
Map-to-model resolution (Å) (0.5 FSC threshold)	4.6	4.2	3.0
Model Composition			
Nonhyrogen atoms	70,048	61,419	77,243
Protein residues	4,182	3,747	4,701
Nucleotides	104	56	103
Ligands	9	9	9
B factor (Å^2)	266	149	89
R.m.s. deviations			
Bond length (Å)	0.003	0.003	0.003
Bond angle (°)	0.553	0.555	0.594
Validation			
MolProbity score	1.8	1.7	1.9
Clash score	7.9	8.1	7.9
Poor rotamers (%)	0	0	0
Ramachandran			
Favored (%)	95.36	95.88	92.49
Allowed (%)	4.52	4.07	7.45
Disfavored (%)	0.12	0.05	0.06

Table S2. Saccharomyces cerevisiae strains

Strain	Genotype	Background	Source
NH0256	*MATa his3Δ1 leu2Δ0 met15Δ0 ura3Δ0*	S288c<br>BY4741	ATCC
DDY4765	*MATa his3Δ1 leu2Δ0 met15Δ0 ura3Δ0 rad16::NATMX*	S288c<br>BY4741	(31)
DDY4776	*MATa his3Δ1 leu2Δ0 met15Δ0 ura3Δ0 elf1::KANMX rad16::NATMX*	S288c<br>BY4741	(31)
NH1356	*MATa his3Δ1 leu2Δ0 met15Δ0 ura3Δ0 rad16::NATMX elf1-85::URA3*	S288c<br>BY4741	This study
CR18	*MATα ura3-52 pep4::HIS3 trp1 his3 leu2 rad7Δ rad26Δ*	BJ5465	(Ding et al., 2010)
WZG508	As CR18, but with plasmid p6FRAD26	BJ5465	This study
WZG510	As CR18, but with plasmid p6FRAD26-631-644/GGG	BJ5465	This study
WZG513	As CR18, but with plasmid p6FRAD26-LKK/AAA	BJ5465	This study
WZG524	As CR18, but with plasmid p6FRAD26-RKR/DDD	BJ5465	This study
WZG525	As CR18, but with plasmid p6FRAD26-LKK/AAA- RKR/DDD	BJ5465	This study
